# Non-CG DNA methylation-deficiency mutations enhance mutagenesis rates during salt adaptation in cultured Arabidopsis cells

**DOI:** 10.1007/s44154-021-00013-2

**Published:** 2021-11-15

**Authors:** Xiaohong Zhu, Shaojun Xie, Kai Tang, Rajwant K. Kalia, Na Liu, Jinbiao Ma, Ray A. Bressan, Jian-Kang Zhu

**Affiliations:** 1 State Key Laboratory of Crop Stress Adaptation and Improvement, School of Life Sciences, Henan University, Kaifeng, China; 2 State Key Laboratory of Cotton Biology, School of Life Sciences, Henan University, Kaifeng, China; 3 Bioinformatics Core, Purdue University, West Lafayette, IN 47907 USA; 4 Department of Horticulture and Landscape Architecture, Purdue University, West Lafayette, IN 47907 USA; 5 Central Arid Zone Research Institute, Jodhpur, 342003 India; 6 State Key Laboratory for Managing Biotic and Chemical Threats to the Quality and Safety of Agro-Products, Institute of Vegetables, Zhejiang Academy of Agricultural Sciences, Hangzhou, 310021 China; 7 State Key Laboratory of Desert and Oasis Ecology, Xinjiang Institute of Ecology and Geography, Chinese Academy of Sciences 830011, Urumqi, China; 8 Shanghai Center for Plant Stress Biology, Shanghai Institutes for Biological Sciences, Chinese Academy of Sciences, Shanghai, 200032 China

**Keywords:** Salt stress, Cell suspension, DNA methylation-deficient mutants, Stress-induced mutagenesis, Stress adaptation

## Abstract

Much has been learned about how plants acclimate to stressful environments, but the molecular basis of stress adaptation and the potential involvement of epigenetic regulation remain poorly understood. Here, we examined if salt stress induces mutagenesis in suspension cultured plant cells and if DNA methylation affects the mutagenesis using whole genome resequencing analysis. We generated suspension cell cultures from two Arabidopsis DNA methylation-deficient mutants and wild-type plants, and subjected the cultured cells to stepwise increases in salt stress intensity over 40 culture cycles. We show that *ddc* (*drm1 drm2 cmt3*) mutant cells can adapt to grow in 175 mM NaCl-containing growth medium and exhibit higher adaptability compared to wild type Col-0 and *nrpe1* cells, which can adapt to grow in only 125 mM NaCl-containing growth medium. Salt treated *nrpe1* and *ddc* cells but not wild type cells accumulate more mutations compared with their respective untreated cells. There is no enrichment of stress responsive genes in the list of mutated genes in salt treated cells compared to the list of mutated genes in untreated cells. Our results suggest that DNA methylation prevents the induction of mutagenesis by salt stress in plant cells during stress adaptation.

**Supplementary Information:**

The online version contains supplementary material available at 10.1007/s44154-021-00013-2.

## Introduction

Soil salinity, caused predominantly by high NaCl levels, has deleterious effects on plant growth (Zhu 2002; Munns and Tester 2008). Salinity triggers osmotic stress and ion cytotoxicity accompanied by nutrient and hormone imbalances and increased production of reactive oxygen species (ROS) (Miller et al. 2010; Jiang et al. 2012). Specific morphological and physiological characteristics enable the survival and growth of salt tolerant plants in highly saline environments. The formation of tolerant features in plants may occur during very long-term challenges of salinity stress, which involves many adaptive changes at not only molecular and cellular levels but also tissue and whole plant levels. At the cellular level, adaption to salt stress can occur more rapidly. For example, suspension cultured cells of several plant species have been shown to adapt to high salt stress in a matter of several months over dozens of cell culture cycles (Binzel et al. 1985). Studies have determined that plants evolved a complex machinery to maintain cellular ion homeostasis by regulating the activity of ion transporters to rapidly respond or acclimate to salt stress (Hasegawa et al. 2000; Zhu 2003). However, the mechanisms underlying adaptation of cells and whole plants to long-term salt stress remain unaddressed.

Successful adaptation depends on phenotypic selection directed toward survival under a specific environmental stress. In plants, either natural or artificial mutagenesis produces genetic variability, which fuels phenotypic selection. Stress-induced mutagenesis (SIM) occurs in both prokaryotes and eukaryotes, including bacteria (Foster 2007), yeasts (Heidenreich 2007), flies (Sharp and Agrawal 2012), human cancer cells (Bristow and Hill 2008), and Arabidopsis plants (Jiang et al. 2014). SIM increases mutation frequencies in response to environmental stress stimuli and plays an important role in stress adaptation (Rosenberg 2001; Tenaillon et al. 2004; Ram and Hadany 2014). Data collected in bacteria suggests that SIM accelerates the speed of adaptation (Bjedov et al. 2003). In Arabidopsis plants, a recent study showed that salt stress increases the frequency of accumulated mutations (Jiang et al. 2014). Over a relatively short timescale of several plant generations, the increased mutations may not be sufficient to cause phenotypic variation. In contrast, epigenetic mechanisms that control gene expression and genomic stability may enable quicker phenotypic variation.

DNA methylation is a conserved epigenetic mechanism in plants and many animals. In Arabidopsis, DNA methylation occurs in three sequence contexts, CG, CHG, and CHH, which are controlled by different pathways. Methyltransferase 1 (MET1) and chromomethylase 3 (CMT3) are required to maintain CG and CHG methylation, respectively, while domains rearranged methyltransferase 2 (DRM2) and CMT2 are important in maintaining CHH methylation (Law and Jacobsen 2010) (Zemach et al. 2013). DRM2 functions through the RNA-directed DNA methylation (RdDM) pathway, which involves two plant specific RNA polymerases, RNA Pol IV (NRPD) and Pol V (NRPE), and 24 nucleotides (24 nt) small interfering RNAs (siRNA). Besides its role in maintaining CHH methylation, the RdDM pathway is critical in de novo DNA methylation in all sequence contexts (Matzke and Mosher 2014). In the *cmt3* mutant, most CHG methylation is abolished with a partial loss of CHH methylation, while both CHG and CHH methylation is greatly reduced in the *drm1 drm2 cmt3* (*ddc*) triple mutant (Furner and Matzke 2011). In the *nrpe1* mutant, CHH methylation at RdDM target regions is abolished. It has not yet been examined if these methylation deficient mutants have different adaptabilities to salt stress. It is possible that the epigenetic effectors that control DNA methylation may alter the accumulation of DNA sequence mutations during long-term salt stress challenges. A previous study suggested that salt stress increases the frequency of accumulated epimutations and DNA sequence mutations in Arabidopsis plants (Jiang et al. 2014), although the mechanisms underlying these mutational responses were not addressed. In mammalian cells, DNA methylation affects the rate and type of single nucleotide substitutions. For example, methylated CpG dinucleotides can promote a high rate of C to T mutation at these sites (Holliday and Grigg 1993). Recent studies demonstrated that the mutation rate of methylated CpG sites is dependent on their methylation levels, although other studies indicated that CpG methylation is not a major determining factor in mutation rates in specific cancer cells (Ossowski et al. 2010; Xia et al. 2012).

Here, we attempted to study the genetic and epigenetic basis of salt adaptation by using suspension-cultured methylation-deficient Arabidopsis cells. The questions we wanted to address include, for example, does salt stress induce genetic mutations in plant cells? Does non-CG DNA methylation affect salt stress induced mutagenesis? Do effectors of DNA methylation affect salt adaptation of plant cells? Our results show that genetic mutations are more highly accumulated over time in salt-treated mutant cells that display better growth under salt stress compared to wild type cells.

## Results

### Methylation deficient mutant cells show high salt adaptability

**Fig. 1:**
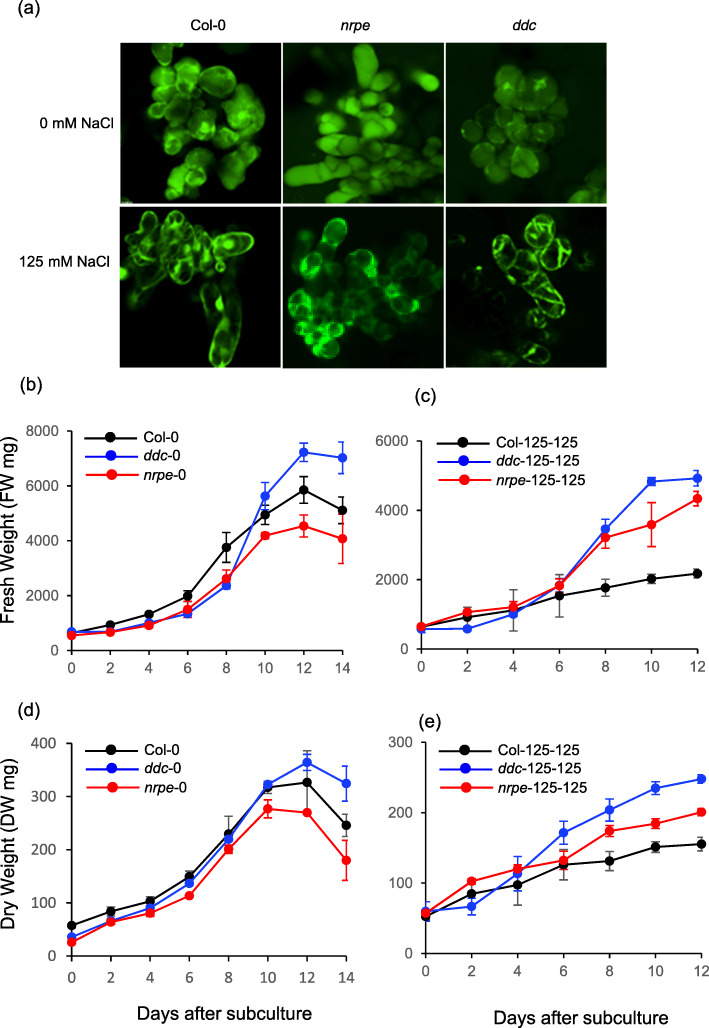
Morphology and growth rates of SAD and SUT cells. **a** FDA staining of SAD and SUT cells. The wild type Col cells (left panel), *nrpe1* cells (middle panel) and *ddc* cells (right panel) were grown in liquid growth medium without (upper panel) and with 125 mM NaCl (lower panel) for approximately 40 cycles of subculture. **b-e** Growth curve of SUT and SAD cells. The SUT and 125 mM NaCl SAD Col cells (black lines), *nrpe1* cells (red line) and *ddc* cells (blue lines) were grown in the medium containing 0 mM NaCl (**b** and **d**) and 125 mM NaCl (**c** and **e**), respectively. Growth curves were measured as an increase of fresh weight (**b** and **c**) and dry weight (**d** and **e**), respectively, as a function of the number of days after subculture in liquid growth medium

To study salt adaptation in plant cells, we generated three lines of suspension cultured Arabidopsis cells from 10-day-old seedlings of the Col-0 wild type (hereafter, Col) and two DNA methylation deficient mutants, *nrpe1* (Stroud et al. 2013) and *ddc* (*drm1 drm2 cmt3)* (Cao and Jacobsen 2002)*.* We subjected the three cell lines to salt stress by stepwise increases in NaCl concentration in the liquid growth medium over a period of 40 cycles of subcultures, starting from 75 mM NaCl to 125 mM NaCl (Fig. S1) to obtain three *s*alt-*ad*apted (named hereafter as SAD) cell lines (Col-125, *nrpe1*–125 and *ddc*-125). Surprisingly, *ddc* cells showed a high adaptive capacity, because they could still grow in medium containing 150 or 175 mM NaCl for more than 20 subculture cycles. In contrast, Col and *nrpe1* cells could survive in medium containing 150 mM NaCl for only a few subculture cycles, and eventually died. So only two additional lines adapted to higher salt were generated, i.e. *ddc*-150 and *ddc*-175. In parallel, three *s*alt untreated (named hereafter as SUT) cell lines (Col-0, *nrpe1*–0 and *ddc*-0) were grown in liquid growth medium without any supplemental NaCl for the same number of subculture cycles. These SUT cell lines served as controls for the SAD cells. We examined the viability of Col-125, *nrpe1*–125 and *ddc*-125 cells together with their control lines at the 40th cycle of subculture. The three SAD cell lines showed a high viability with no obvious morphological changes except for a higher proportion of cells with cytoplasmic vacuolization (Fig. 1a), which can be an adaptive physiological response (Henics and Wheatley 1999).

Next, we compared the growth rates of SAD lines (cell-125) with their control SUT lines (cell-0). With a similar volume of starting cells, Col-0, *nrpe1*–0 and *ddc*-0 cell lines showed similar growth rates in exponential phase but with different timing of stationary phase. The *nrpe1*–0 cells reached stationary phase earlier than Col-0 and *ddc*-0 cells, and *ddc*-0 cells reached stationary phase a little later, but with higher mass when measured by both fresh and dry weight (Fig. 1b, d). Compared to the SUT cells, cells grown in 125 mM NaCl-containing medium showed slower growth with a prolonged exponential phase. Interestingly, *nrpe1*–125 and *ddc*-125 cells grew faster with higher fresh and dry weight in exponential phase than Col-125 cells when grown in 125 mM NaCl-containing medium (Fig. 1c, e). These results suggest that within the same duration of salt stress exposure as wild type Col cells, the methylation deficient cells (*nrpe1* and *ddc*) developed better adaptation to salt stress.

### Mutagenesis is enhanced by salt stress in methylation deficient mutant cells

To investigate if salt induced mutagenesis operates in the wild type and methylation deficient cells, we used whole-genome sequencing (20-50X genome coverage) (Table S1) to identify genetic mutations in both SAD (Col-125, *nrpe1*–125 and *ddc*-125) and SUT cell lines (Col-0, *nrpe1*–0 and *ddc*-0), as well as early generation cells (Col-E, *nrpe1*-E and *ddc*-E) from which cell-0, cell-125, cell-150 and cell-175 were derived (Fig. S1). The early generation cells only experienced two cycles of subcultures after they were generated from 10-day-old seedlings, so they served as a reference for SAD and SUT cells. For the *ddc* cells, 150 mM and 175 mM NaCl SAD cell lines (*ddc*-150 and *ddc*-175) were also included in the analysis to examine how the severity of salt stress affects mutagenesis.

We detected a total of 1143 DNA sequence mutations in SAD cells and 381 in SUT cells. The mutations consisted of 1357 single-base substitutions (SBSs) and 167 short insertion and deletion (Indel) mutations (Table 1, Table S2 and Table S3; accession number PRJNA766713). Potential large structural variations were not analyzed because they are difficult to detect by Illumina sequencing. For *nrpe1* and *ddc* cells, the total numbers of mutations were approximately two-fold higher in SAD cells than in SUT cells. While the total indel mutations in Col-125 cells appeared similar to Col-0, the number of SBS mutations appeared lower in Col-125 cells compared to Col-0 cells (Table 1). We estimated the mutation rate as the number of mutations per site per unit time (time = days of each cycle multiples the number of subculture cycles), because the number of generations of cultured cells could not be precisely determined. As a result, SBS and indel mutation rates in *ddc*-125, 150 and 175 cells were more than two-fold of that in *ddc*-0, while *nrpe1*–125 cells showed about a 1.5-fold higher SBS mutation rate but a similar indel mutation rate, compared to *nrpe1*–0 cells. There was a decrease in SBS mutation rate in Col-125 cells compared to Col-0 cells, although their indel mutation rates were similar. These results showed that the overall mutation rates increased in SAD methylation deficient mutant cells, but not in the wild type cells, suggesting that DNA methylation prevents the induction of mutagenesis by salt stress in plant cells during stress adaptation.

**Table 1:** Number of mutations and their distributions among functional classes within the genome of 1st batch of SAD and SUT cells

	Col	Col	*nrpe*	*nrpe*	*ddc*	*ddc*	*ddc*	*ddc*
NaCl (mM)	0	125	0	125	0	125	150	175
SBS								
Coding	13	11	25	40	19	55	51	58
IG	33	20	36	53	20	63	55	65
Intron	6	9	9	19	11	26	24	28
TE	40	41	40	71	53	97	98	88
UTR	2	1	4	8	2	9	12	9
ncRNA	2	0	6	3	1	2	1	2
pseudogene	2	1	1	3	1	3	3	2
Total Number	98	83	121	197	107	255	244	252
MR (X10^−10^)	28.02	18	26.18	41.7	25.35	55.33	53.09	54.62
INDEL								
Coding	0	0	0	0	0	0	0	0
IG	13	16	11	7	4	8	5	10
Intron	5	6	3	4	1	2	3	1
TE	4	4	4	7	3	8	6	9
UTR	2	2	3	3	1	2	3	4
ncRNA	0	0	0	0	0	0	0	0
pseudogene	0	0	1	1	0	0	0	0
Total Number	24	28	22	9	9	20	17	24
MR (X10^−10^)	6.86	6.07	4.76	4.86	2.13	4.34	3.7	5.2

*Note*: *MR* (Mutation Rate): The mutation rate was calculated as the number of mutations per site per unit time (time = days of each cycle multiples the number of subculture cycles) because the number of generations of cultured cells could not be precisely determined

To confirm the difference in mutation rates in the cell lines, we generated independently a second batch of SAD Col, *nrpe1* and *ddc* cell lines. These cell lines were stepwise adapted to the highest salt concentration, 150 mM NaCl-containing growth medium through a total of 20 subculture cycles. SAD cell lines were grown in 150 mM NaCl-containing medium for six subculture cycles when cells were harvested for genome resequencing analysis. Although the total numbers of SBSs and indels were much smaller than the ones detected in the first batch of cells as described above, again the total numbers of mutations were higher in SAD cells than the ones in SUT cells for the *nrpe1* and *ddc* mutants (Table S4). Especially for *ddc* cells, SBS and indel mutation rates were 3.09 and 4.66 fold higher in *ddc*-150 than the ones in *ddc*-0 cells, respectively (Table S4). No increased SBS and indel accumulation was detected in Col-150 cells compared to Col-0, and SBS and indel mutation rates were similar between Col-0 and Col-150 cells. For unknown reasons, the overall SBS and indel mutation rates of Col cells were higher than *nrpe* and *ddc* cells in this second batch of cell lines. Presumably, these mutations may be related to the Col-0 used in this study, but are not related to salt treatment since total mutation numbers of salt-treated wild type cells was similar to salt untreated Col-0 wild type cells. Together, these results support the conclusion from the first batch cells that mutagenesis is enhanced by salt stress in DNA deficient mutant cells but not in wild type cells.

### Salt induced mutations have distinctive spectra in methylation deficient mutants

**Fig. 2:**
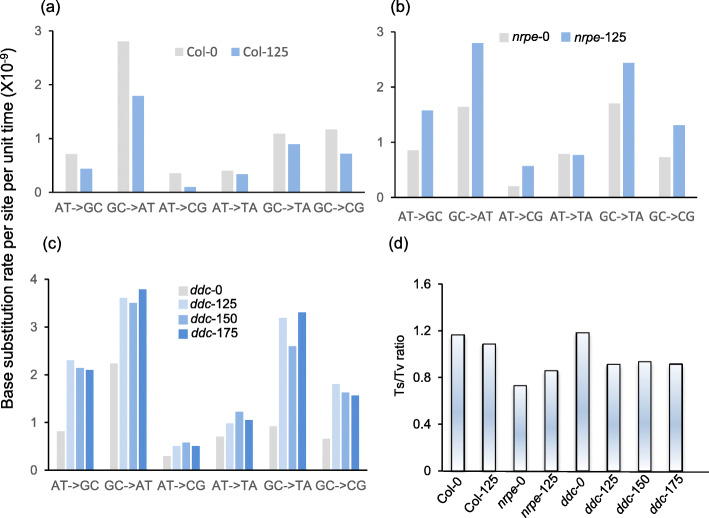
Spectrum of single base substitution mutations. Rates of single base transitions (AT-GC and GC-AT) and transversions (AT-CG, AT-TA, GC-TA and GC-CG) were measured in SUT and SAD Col (**a**), *nrpe1* (**b**) and *ddc* (**c**) cells. Transition to transversion ratios were calculated for SAD and SUT Col, *nrpe1* and *ddc* cells (**d**)

To characterize the spectrum of mutations that occur in the cells, we first calculated the rate of six different types of base substitution and the ratio of transition and transversion SBSs (Ts/Tv) for the first batch of cells. Overall, both transition and transversion rates were increased in SAD *nrpe1* and *ddc* cells, but were reduced in SAD Col-0 cells (Fig. 2a, b, c). Ts/Tv ratios were slightly lower in Col-125 and *ddc-*125 cells than in Col-0 and *ddc*-0, but was slightly higher in *nrpe*1–125 cells than in *nrpe1*–0 cells. Apparently increasing salt intensity had no effect on the ratio of Ts/Tv in SAD *ddc* cells, because Ts/Tv ratios were similar among *ddc*-125, *ddc*-150 and *ddc*-175 (Fig. 2d). For SUT cells, Ts/Tv ratios of Col-0 and *ddc*-0 cells were 1.17 and 1.18, respectively, indicating that the transversion of SBSs was relatively high in *nrpe1*–0, in which the Ts/Tv ratio was 0.73 (Fig. 2d). Next, we calculated the Ts/Tv ratios of the second batch of SAD and SUT Col, *nrpe* and *ddc* cell lines. Similar to the ones detected in the first batch of cell lines, Ts/Tv ratio of Col-150 was slightly lower than that of Col-0 cells, but for *ddc*-150 cells, Ts/Tv ratio was greatly reduced compared to *ddc*-0 cells (Fig. S2). In contrast, Ts/Tv ratio was higher in *nrpe*-150 cells than in *nrpe*-0 cells (Fig. S2). Taken together, the ratios of Ts/Tv in both batches of cultured cells are much lower than the one reported in MA lines in Arabidopsis plants (Ossowski et al. 2010; Jiang et al. 2014). The findings of increased transversion frequency in SAD Col and *ddc* cells are similar to that found in a MA line grown in saline soil for 10 successive generations (Jiang et al. 2014).

**Fig. 3:**
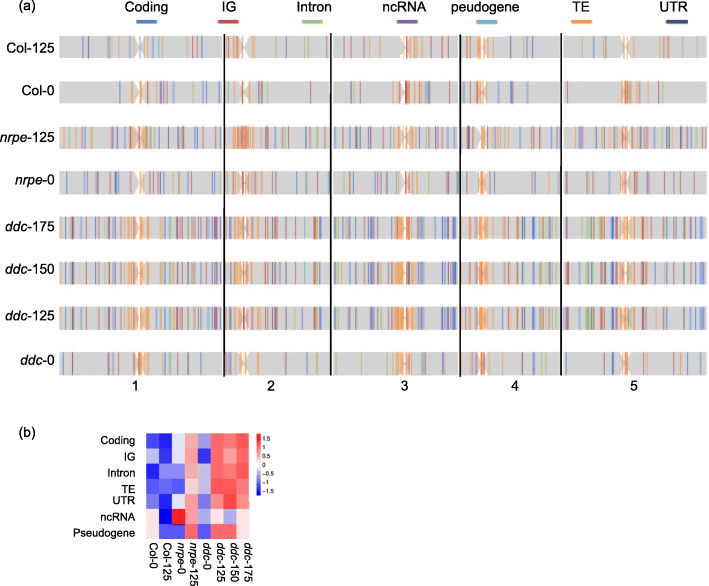
Chromosomal distribution of SBS mutations. **a** Colored lines represent mutations that occurred in genomic regions of SUT and SAD Col, *nrpe1* and *ddc* cells with different functional types indicated on the top of the image. **b** Heatmap of SNP numbers within genomic regions of SUT and SAD Col, *nrpe1* and *ddc* cells

Next, we analyzed the distribution of SBSs and indels mutations of the first batch of cells in five chromosomes. As shown in Fig. 3a, near the pericentromeric and/or centromeric region, there were high enrichments of TE mutations in salt adapted *nrpe1* and *ddc* cells, but not in Col-125 cells. High TE mutations occurred near the pericentromere, while most mutations within the coding region were detected in chromosome arms in all three SAD *ddc* cell lines. The large number of TE mutations may account for the high mutation rates near the centromere in both SAD and SUT Col, *nrpe1* and *ddc* cells (Table 1). No chromosome-specific increased or decreased mutation rates were observed in SAD and SUT Col, *nrpe1* and *ddc* cells (Fig. S3). Mutation rates of all five chromosomes increased in SAD *nrpe1* and *ddc* cells, but not in SAD Col cells (Fig. S3). We further categorized all mutations into seven different functional categories. As shown in Table 1, SBSs in *ddc*-0 cells were 17.76 (19/107), 18.69 (20/107) and 1.87% (2/107) of the total mutations in coding, intergenic and untranslated regions (UTR), respectively. However, the average percentage of SBSs in *ddc*-125, 150 and 175 cells were 21.57 (55/255), 20.90 (51/244) and 23.02% (58/252) in coding, 24.71 (63/255), 22.54 (55/244) and 25.79% (65/252) in intergenic region, and 3.53 (9/255), 4.92 (12/244) and 3.57% (9/252) in untranslated regions, respectively, indicating a relatively higher enrichment in these three regions in *ddc*-125, 150 and 175 cells than those in *ddc*-0 cells (Fig. 3b). In contrast, high percentages of SBSs were found in intron, TE, UTR and pseudogene, but not in coding or intergenic regions of *nrpe1*–125 cells. SBSs in intron, TE, UTR and pseudogene were 9.64 (9/121), 36.04 (40/121), 4.06 (4/121) and 1.52% (1/121) in *nrpe1*–125 cells and 7.43 (19/197), 33.05 (71/197), 3.30 (8/197) and 0.83% (3/197) in *nrpe1*–0 cells, respectively (Table 1). No specific enrichments of SBSs and indels in seven functional categories were observed in Col-125 cells compared to Col-0 cells except for TE, in which the percentage of SBSs were 49.40% in Col-125 cells and 40.82% in Col-0 cells, showing relatively high percentages of SBSs in TEs of Col-125 cells. Overall, the total number of indels was smaller than SBSs in seven functional categories, and no indel mutations were found in the coding region of all SAD and SUT cells. Instead, indels mostly occurred in intergenic regions, intron, TE and UTR. It appeared that indel mutation rates were higher without special enrichment in certain regions in *ddc*-125, 150 and 175 cells, while they were similar between Col-0 and Col125 cells, and between *nrpe1*–0 and *nrpe1*–125 cells. The *nrpe1*–125 cells also showed enriched indels in the TE region. Again, for wild type Col-0 cells, no difference in the total number of indel mutation or indel mutation rates was detected between Col-0 and Col-125 cells.

We also estimated the effects of the selection acting on the protein coding sequence by calculating frequencies of nonsynonymous and synonymous mutations (see method). Ka/Ks is the ratio of the number of nonsynonymous substitutions per non-synonymous site (K_a_), in a given period of time, to the number of synonymous substitutions per synonymous site (K_s_), in the same period. As shown in Table 2, for the first batch of cells, Ka/Ks for SAD cells were more than 1 except for *nrpe1*–125 cells, suggesting that cultured cells are mostly under strong positive selection when subjected to salt stress. Next, we used the program SIFT 2.1.2 (Ng and Henikoff 2001, Ng and Henikoff 2003) to determine deleterious (nsSNP-d) and tolerated nonsynonymous mutations (nsSNP-t) based on the location of mutations in the protein structure (Gunther and Schmid 2010). We measured the relative frequency of deleterious mutations by calculating the ratio of deleterious to total nonsynonymous mutations (nsSNP-d/nsSNP). We found that more than 50% of nsSNPs were predicted to be deleterious in the first batch of the cultured cells, a much higher rate than those reported in Arabidopsis and rice plants (less than 0.25 in Arabidopsis and rice) (Gunther and Schmid 2010) (Table 2). Further, the ratios of deleterious nsSNPs were relatively high in salt adapted Col and *ddc* cells compared to their SUT cells, while it was similar between *nrpe1*–0 and *nrpe1*–125 cells. Our results indicate that nsSNPs were biased by selection in the cultured cells, among which around half were deleterious nsSNPs. In addition, salt stress slightly increased the accumulation of deleterious nsSNPs in the first batch of Col and *ddc* cells that were adapted to salt stress in a long period of time.

**Table 2:** Relative proportion of deleterious nsSNPs in the first batch of SAD and SUT cells

Cell line	Ka/Ks	nsSNP-d	nsSNP-t	nsSNP	nsSNP-d/nsSNP
Col-0	0.3	6	3	9	0.67
Col-125	> 1	7	2	9	0.78
*nrpe*-0	1.32	15	4	19	0.79
*nrpe*-125	0.69	23	8	31	0.74
*ddc*-0	3.52	8	8	16	0.5
*ddc*-125	1.47	27	17	44	0.61
*ddc*-150	2.21	24	17	41	0.59
*ddc*-175	1.94	29	16	45	0.64

*Note*: Ka/Ks is the ratio of the number of nonsynonymous substitutions per non-synonymous site (K_a_), in a given period of time, to the number of synonymous substitutions per synonymous site (K_s_), in the same period. nsSNP-d is deleterious mutations, and nsSNP-t is tolerated nonsynonymous mutations based on the location of mutations in the protein structure. nsSNP-d/nsSNP is the ratio of deleterious to total nonsynonymous mutations

### Mutations of stress responsive genes are not enriched in SAD compared to SUT *ddc* and *nrpe1* cells

**Fig. 4:**
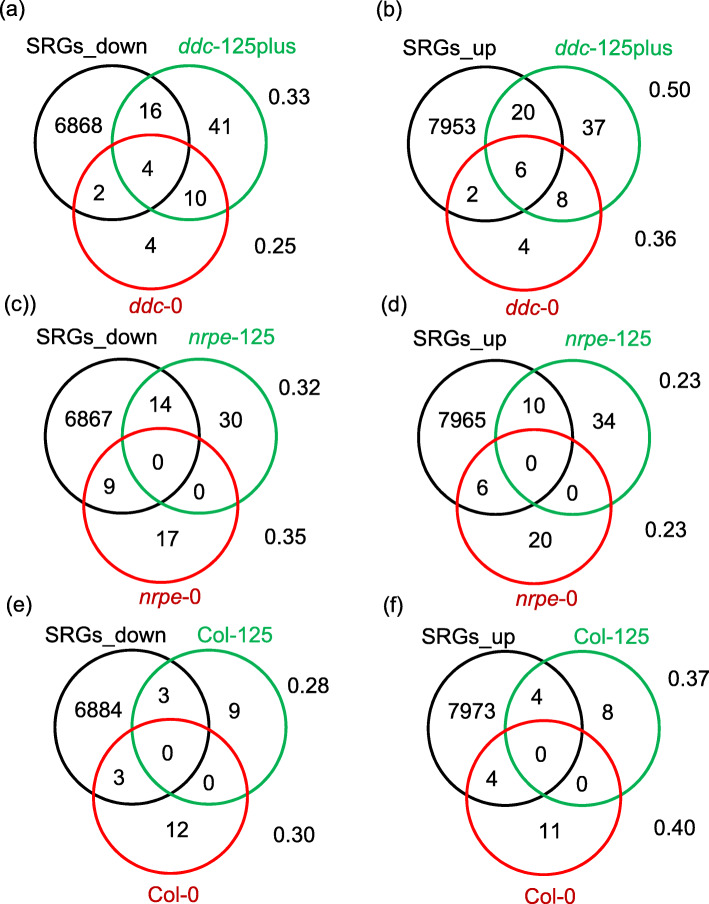
Classification of non-synonymous mutations in SUT and SAD cells for stress responsive genes. Non-synonymous mutations were identified as down- and up-regulated stress responsive genes for SUT and SAD *ddc* (**a** and **b**), *nrpe1* (**c** and **d**) and Col (**e** and **f**) cells. For SAD *ddc* cells, non-synonymous mutations detected in *ddc*-125, 150 and 175 cells are combined and presented as *ddc*-125 plus. Numbers near the diagrams show the ratio of responsive genes to the total non-synonymous genes in SUT or SAD cells. SRGs: stress responsive genes

To determine if salt induced mutagenesis is directed toward specific genes involved in the control of salt tolerance, we counted the number of mutations in SAD and SUT cell lines that occurred in stress responsive genes (SRGs). We used three databases to collect the responsive genes to salt, drought, osmotic stress and ABA. These three databases are 1) RARGE (the RIKEN Arabidopsis Genome Encyclopedia) database (Akiyama et al. 2014), 2) DRASTIC (Database Resource for the Analysis of Signal Transduction In Cells (Button et al. 2006), and 3) microarray data deposited in GEO datasets to identify stress responsive genes using GEO2R (Barrett et al. 2013). For the RARGE database, we extracted genes that have a ≥ 2.5-fold change (FC) of expression at any time point under different stresses (drought, NaCl, and ABA, and rehydration after 2 h dehydration). For the DRASTIC and GEO databases, stress responsive genes were identified with a *P*-value < 0.01 and |log_2_(FC)| > 2. Salt and dehydration stress responsive genes from three pools were classified into two categories, down- and up-regulated genes (Supplemental Data Set1). For the first batch of cells, among total coding region mutations (SBScds), 20 and 26 were found to be in down or up regulated genes in SAD *ddc* cells (*ddc*-125, *ddc*-150 *ddc*-175) and 14 and 10 in *nrpe1*–125 cells, respectively (Fig. 4a, b, c, d; Table S5). Total SRGs were much higher in SAD *ddc* and *nrpe1* cells than those in SAD Col cells, in which down and up regulated genes were 3 and 4 respectively (Fig. 4e and f; Table S5). However, the ratios of SRGs to SBScds were slightly higher in Col-125 than Col-0 cells, while they were similar between SAD and SUT *ddc* and *nrpe1* cells. Analysis of the first batch of cells reveals that there were no specific increases in mutations in stress responsive genes in SAD cells, suggesting that salt stress induced mutagenesis is not directed to specific sites related to salt tolerance. Rather, the increase in the total number of mutations in stress responsive genes in SAD cells may provide more genetic variation to provide a selective advantage by facilitating salt tolerance.

## Discussion

Here, we demonstrated that salt induced mutagenesis in cultured cells is affected by regulators of DNA methylation. Salt induced mutagenesis increased the frequency of accumulated mutations with distinctive spectrum and patterns in two non-CG DNA methylation deficient mutant cell lines. Intriguingly, both DNA methylation deficient mutant cell lines showed high adaptability to salt stress. These findings provide insights into epigenetic regulation during stress adaptation and genomic evolution.

### High mutation rate may offer an adaptive advantage

The ability to adapt through mutational mechanisms is central to bacterial pathogenicity and to tumorigenesis of cancer cells. It has been established very early that enhanced mutagenesis is associated with the tissue culture of plant cells (Braun 1959). However, we know little about the mechanisms of stress induced mutagenesis underlying stress adaptation in plant cells. There can be preexisting variation as in chimeric cultures, induced by chromosomal aberrations and errors created during the cell cycle. In addition, transposable element activation has been identified as one of the mechanisms of cell culture mutagenesis (Azman et al. 2014; Larkin et al. 1981). In this study, we established three salt-adapted Arabidopsis cell lines: wild type Col and two DNA methylation deficient mutants, *ddc* and *nrpe1*, in which a large reduction in genome-wide non-CG methylation has been documented (Stroud et al. 2013). Using whole genome resequencing data, we found that salt stress increased the frequency of mutations in *ddc* and *nrpe1* cells, but not in Col wild type cells, suggesting that non-CG methylation regulates salt stress induced mutagenesis in cultured Arabidopsis cells. We propose that salt stress elevates mutation frequency to promote a high accumulation of mutations for adaptive advantage. Salt stress induced mutagenesis may operate preferentially in specific genomic regions in different cell lines. Although we have no evidence that salt stress induced mutagenesis is directed to specific gene loci that may confer a selective advantage based on the analysis of stress responsive genes, we observed a correlation between the higher mutagenesis rate and better adaptability. Salt stress caused a 1.5–2 fold increase in mutation rate with a relatively high frequency in coding and regulatory regions, such as UTRs and intergenic regions, in SAD *ddc* cells, and in TE and intronic regions in SAD *nrpe1* cells. The ratio of SRGs to SBScds did not appear high in SAD *ddc* and *nrpe1* cells, suggesting that salt induced mutagenesis is not directed to specific stress responsive genes. However, overall increased SRGs may create more adaptive variants for selective advantage by facilitating salt tolerance, which can enable the development of adaptation and perhaps stable tolerance due to genetic assimilation. The high frequency of mutations in UTRs may contribute to adaptation since UTRs play important regulatory roles in gene expression (Srivastava et al. 2018). On other hand, the high percentage of deleterious mutations may result from relaxed purifying selection due to heterogeneous effects of deleterious mutations in cultured cells which were maintained during mitosis or lost during subcultures. In fact, mutation rates of SAD cells are underestimated when measured as the number of mutations per site per unit time instead of per generation in this study, because SAD cells grow more slowly than SUT cells (Fig. 1) (Binzel et al. 1985).

### DNA methylation regulates salt stress induced mutagenesis

Whether and how DNA methylation affects stress-induced mutagenesis is an area of intense interest. Studies suggest that spontaneous deamination of methylated cytosine is a major source of SBS mutations. In mammals, deamination rate of methylated cytosine is more than 2 fold greater than unmethylated cytosine (Ehrlich et al. 1986). CpG substitution rate is significantly correlated with methylation levels, but negatively correlated with GC content (Mugal and Ellegren 2011). However, in Arabidopsis studies show that high GC-AT transition is unrelated to the level of CpG methylation but rather is attributed partially to UV induced mutagenesis (Ossowski et al. 2010). Our findings of high rates of induced mutations in *nrpe1* and *ddc* cells under salt stress are very intriguing. Previous studies demonstrated that the vast majority of non-CG, but not CG, methylation is eliminated in *nrpe1* and *ddc* mutant plants (Stroud et al. 2013; Zhang et al. 2013). Relatively high GC-AT transitions occurred in Col-0 cells but were low in *nrpe1*–0 and *ddc*-0 cells, suggesting that the level of non-CG methylation in cultured cells may affect GC substitution to some extent. Under salt stress, SBS mutations in *nrpe1* and *ddc* cells were attributed to increases in both transition and transversion rates. But GC-TA and GC-CG transversion in SAD *ddc* cells increased by 3–6 fold, resulting in a decreased Ts/Tv ratio, while transversions in both SAD and SUT *nrpe1* cells were higher than transition, and the resulting Ts/Tv ratios were less 1. Another explanation for the relatively low Ts/Tv ratio in cultured cells is that UV induced mutations may be limited in cells cultured in the dark. Nevertheless, how non-CG methylation affects salt induced mutagenesis on GC transversion requires further investigation. In addition, a high density of TE mutations near the pericentromeric and/or centromeric region is a unique signature of SAD *nrpe1* and *ddc* cells. Further, a relatively high accumulation of mutations occurred in coding, intergenic and untranslated regions in SAD *ddc* cells and in TE and intronic region in SAD *nrpe1* and Col cells. These patterns may correlate with alterations in non-CG methylation levels in *ddc* and *nrpe1* cells under salt stress, but it is unclear if they result from the generation and/or the selection of mutations under salt stress. We hypothesize that mutations in cultured cells are not strongly affected by purifying selection because of their heterogenous effects, but nonsynonymous mutations are strongly biased by positive selection.

### Mechanism underlying salt stress induced mutagenesis in cultured cells

The molecular mechanisms underlying salt inducible mutagenesis is most likely a heterogeneous process in cultured cells, implying that it is not a single or simple process. Suspension-cultured cells are subjected to osmotic and hormone stress, which along with pre-existing and mitotic based errors may account for mutations detected in SUT cells. Adaptation of plant cells to different culture environments may initiate changes in cell differentiation programs preexisting or induced in a cell culture. Thus, differentiation based changes in gene expression including changes in chromatin structure may underlie phenotypic adaptation to culture conditions.

However, no similar types of SBScds were detected in the SAD and SUT Col and *nrpe1* cells. In *ddc* cells, about 20% of the mutations occurred in both SAD and SUT *ddc* cells. Out of the total mutations including SBSs and indels, only 1.12 and 2.91% occurred in both SAD and SUT Col and *nrpe1*, respectively (Fig. S4), suggesting that mutations in SAD cells are generated mostly by salt induced mutagenesis. Salt stress is generally accompanied by oxidative stress, which causes DNA damage. To elicit mutations, input could be either DNA damage or replication error, whereas output should be determined by mismatch repair pathways and fitness effects of the mutations (Rosenberg 2001; Baer et al. 2007). First, apparent differences in the molecular spectrum of mutations between SAD and SUT cells likely occur from variations in cellular mutagenicity and/or proofreading of DNA replication, implying that error-prone DNA polymerases are actively involved in salt stress induced mutagenesis. Some error-prone DNA polymerases are error–free when replicating the T-T dimer but may be error-prone when replicating C-C dimers (Ohashi et al. 2000). Therefore, various translesion polymerases with different accuracy rates may bypass DNA lesions to create a wide spectrum of mutations in specific cell types. High mutation rates under salt stress in *nrpe1* and *ddc* cells may also be due to less efficient mismatch repair in *nrpe1* and *ddc* cells than in Col cells under salt stress.

## Materials and methods

### Generation of suspension cell lines

Arabidopsis wild type Col-0 and DNA methylation deficient mutants, *nrpe1* and *ddc*, were used to generate cell suspension cultures as previously described (Encina et al. 2001). Plant seeds from three genotypes were sterilized with 10% bleach plus 0.01% Trion-100 and planted on 0.6% Agar plates containing full strength of MS salt, respectively. Approximately one gram of 10-day-old-seedlings were chopped finely and put into 25 ml liquid cell suspension medium (full strength MS salt containing vitamin and 3% sucrose) in a 50 ml Erlenmeyer flask. Flasks were placed in an orbital shaker set at 100 rpm in darkness. After 10 days, cultures were filtered through a 100um mesh to remove tissue pieces. Homogenized cell suspensions were subcultured every 10 days, and subjected to salt stress by adding 1 M NaCl prepared in the culture medium to yield stepwise final concentrations of 75 mM, 100, 125, 150, and 175 mM NaCl. SAD cells were kept in the medium containing a given concentration of NaCl for about 3–5 cycles of subcultures before subjecting the cells to the medium containing a higher concentration of NaCl (Fig. S1). Because SAD cells grow more slowly than SUT cells, 2 ~ 3 fold higher volume of SAD cells were taken for every subculture cycle to keep both SAD and SUT cell lines subcultured every 10 days.

### Measurement of cell growth rate and viability

Cultured cells from a 200 ml Erlenmeyer flask were divided equally into smaller flasks, each containing 25 ml cell culture. Three flasks of cells for each cell line were harvested at 0, 2, 4, 6, 8, 10, 12 and 14 days after subcultures. Fresh weight and dry weight of cells were measured after harvest. Morphology and viability of cells were examined by the FDA staining method (Amano et al. 2003). Images were acquired under epifluorescence microscope.

### Analysis of re-sequencing data

Genomic DNA from each cell line was extracted and sequenced using libraries from a ~ 100 bp fragments. Raw reads were aligned to the Arabidopsis genome (TAIR10) using the BWA-MEM program. SAMtools software was used to sort and convert the alignment into bam files (Li et al. 2009). The Picard tool (http://picard.sourceforge.net/) removed duplicated reads due to PCR amplification.

### Identification and classification of SNPs and indels

To determine the purity of the cells with mutations after multiple subculture generations, we used Virmid to estimate the contamination level (a, 0 ≤ a ≤ 1) of the samples (Kim et al. 2013). With contamination level as one input, we used VarScan2 to call small variants (SNPs and indels) (Koboldt et al. 2012). Specifically, ‘samtools mpileup’ generated pileup files for each paired samples (samples with and without salt stress). Then we used ‘VarScan.v2.3.7.jar somatic’ to identify SNPs and indels. To annotate and predict the effects of variants on genes, we used snpEff (Cingolani et al. 2012) to report putative variant impact on each variant (SNPs and indels).

Genomic regions were divided into different classes based on annotated genome information: coding, intron, UTR, ncRNA, pseudogene, transposable elements (TE) and intergenic regions (IG): We grouped all analyzed sites into the functional classes with a priority of Coding > Intron > UTR > ncRNA > pseudogene > TE > IG.

Based on the degree of conservation of amino acid residues in the alignments results derived from homologous protein sequences, SIFT can predict whether an amino acid substitution caused by SNP affects protein function. Homologous protein sequences were identified by PSI-BLAST (John and Sali 2004) in SIFT. We used the UniRef90 protein database for homolog identification. If a non-synonymous SNP could affect protein function, this SNP was classified as deleterious for protein functions (Ng and Henikoff 2003).

### Stress responsive genes

We used the following three databases to determine genes responsive to salt, drought, osmotic and ABA stress: 1) The RARGE (the RIKEN Arabidopsis Genome Encyclopedia) database (Akiyama et al. 2014) used extracted genes that showed a ≥ 2.5-fold expression change at any time course under different stresses (drought, NaCl, and ABA, and rehydration after 2 h dehydration), 2) DRASTIC (Database Resource for the Analysis of Signal Transduction In Cells (Button et al. 2006) and 3) microarray data deposited in GEO datasets identified stress responsive genes using GEO2R (Barrett et al. 2013).

## Supplementary Information

**Additional file 1: Figure S1.** Generation of SAD cell suspension lines. (a). Arabidopsis seedlings were grown on MS plates. (b). Ten days-old-seedlings were finely chopped and cultured in the liquid growth medium for ~ 10 days. (c). Suspension cells were obtained by filtering the cultures. (d). Cell lines were subjected to stepwise increase in NaCl concentration in the growth medium. The number within the box indicates the number of subculture (sbc) in given salt treatment before the cells were shifted to the next subculture with increased salt concentration. The number next to the box indicates the total number of subcultures (SBC) in given salt treatment when the samples were collected for the analysis. (e). SAD cell lines were established over subculture cycles. (f). Growth curves were measured starting from three replicates for each cell line. **Figure S2.** Transition to transversion ratios of second batch of cell lines. Ts/Tv ratios were calculated for second batch of SAD and SUT Col, *nrpe1* and *ddc* cells. **Figure S3.** Chromosome-specific mutation rates. Mutation rate per site per unit time was calculated for each chromosome-specific mutation rate based on the mutation site on each of five chromosomes of SUT and SAD Col (a), *nrpe1* (b) and *ddc* (c) cells. **Figure S4.** Total mutations in SAD and SUT cells. Number and overlap of total SBSs and indels were detected in SAD and SUT Col (a), *nrpe1* (b) and *ddc* (c) cells.

**Additional file 2: Table S1.** Number of mutations and their distributions among functional classes within the genome of 1^st^ batch of SAD and SUT cells. **Table S2.** Relative proportion of deleterious nsSNPs in the first batch of SAD and SUT cells. **Table S3.** A list of total insertion and deletion (Indel) mutations in the first batch of SAD and SUT cells. **Table S4.** Number of mutations and their distributions among functional classes within the genome of 2^nd^ batch of SAD and SUT cells. **Table S5.** A list of total coding region mutations in the first batch of SUT and SAD cells.

**Additional file 3.**

## Data Availability

The data supporting our findings can be found in the supplementary materials.
