# Publisher Correction: Non-CG DNA methylation-deficiency mutations enhance mutagenesis rates during salt adaptation in cultured Arabidopsis cells

**DOI:** 10.1007/s44154-021-00017-y

**Published:** 2021-12-02

**Authors:** Xiaohong Zhu, Shaojun Xie, Kai Tang, Rajwant K. Kalia, Na Liu, Jinbiao Ma, Ray A. Bressan, Jian-Kang Zhu

**Affiliations:** 1 State Key Laboratory of Crop Stress Adaptation and Improvement, School of Life Sciences, Henan University, Kaifeng, China; 2 State Key Laboratory of Cotton Biology, School of Life Sciences, Henan University, Kaifeng, China; 3 Bioinformatics Core, Purdue University, West Lafayette, IN 47907 USA; 4 Department of Horticulture and Landscape Architecture, Purdue University, West Lafayette, IN 47907 USA; 5 Central Arid Zone Research Institute, Jodhpur, 342003 India; 6 State Key Laboratory for Managing Biotic and Chemical Threats to the Quality and Safety of Agro-Products, Institute of Vegetables, Zhejiang Academy of Agricultural Sciences, Hangzhou, 310021 China; 7 State Key Laboratory of Desert and Oasis Ecology, Xinjiang Institute of Ecology and Geography, Chinese Academy of Sciences, Urumqi, 830011 China; 8 Shanghai Center for Plant Stress Biology, Shanghai Institutes for Biological Sciences, Chinese Academy of Sciences, Shanghai, 200032 China

**Correction to: Stress Biol 1, 12 (2021)**

**https://doi.org/10.1007/s44154-021-00013-2**

Following publication of this article (Zhu et al. 2021), it is noticed that the article contained an error. The affiliation of the author Ray A. Bressan should be below:

4. Department of Horticulture and Landscape Architecture, Purdue University, West Lafayette, IN 47907, USA.

The author Ray A. Bressan’s affiliation has been updated in this Correction, and the original article has been updated as well.
