# Supplementary material for: Non-CG DNA methylation-deficiency mutations enhance mutagenesis rates during salt adaptation in cultured Arabidopsis cells
Source: Stress Biol. 2021 Nov 15;1(1):12. doi: 10.1007/s44154-021-00013-2 (PMC10441993; doi:10.1007/s44154-021-00013-2)
Supplement: Supplementary file 1 — Additional file 1: Figure S1. Generation of SAD cell suspension lines. (a). Arabidopsis seedlings were grown on MS plates. (b). Ten days-old-seedlings were finely chopped and cultured in the liquid growth medium for ~ 10 days. (c). Suspension cells were obtained by filtering the cultures. (d). Cell lines were subjected to stepwise increase in NaCl concentration in the growth medium. The number within the box indicates the number of subculture (sbc) in given salt treatment before the cells were shifted to the next subculture with increased salt concentration. The number next to the box indicates the total number of subcultures (SBC) in given salt treatment when the samples were collected for the analysis. (e). SAD cell lines were established over subculture cycles. (f). Growth curves were measured starting from three replicates for each cell line. Figure S2. Transition to transversion ratios of second batch of cell lines. Ts/Tv ratios were calculated for second batch of SAD and SUT Col, nrpe1 and ddc cells. Figure S3. Chromosome-specific mutation rates. Mutation rate per site per unit time was calculated for each chromosome-specific mutation rate based on the mutation site on each of five chromosomes of SUT and SAD Col (a), nrpe1 (b) and ddc (c) cells. Figure S4. Total mutations in SAD and SUT cells. Number and overlap of total SBSs and indels were detected in SAD and SUT Col (a), nrpe1 (b) and ddc (c) cells. [file 44154_2021_13_MOESM1_ESM.pptx]

## Slide 1
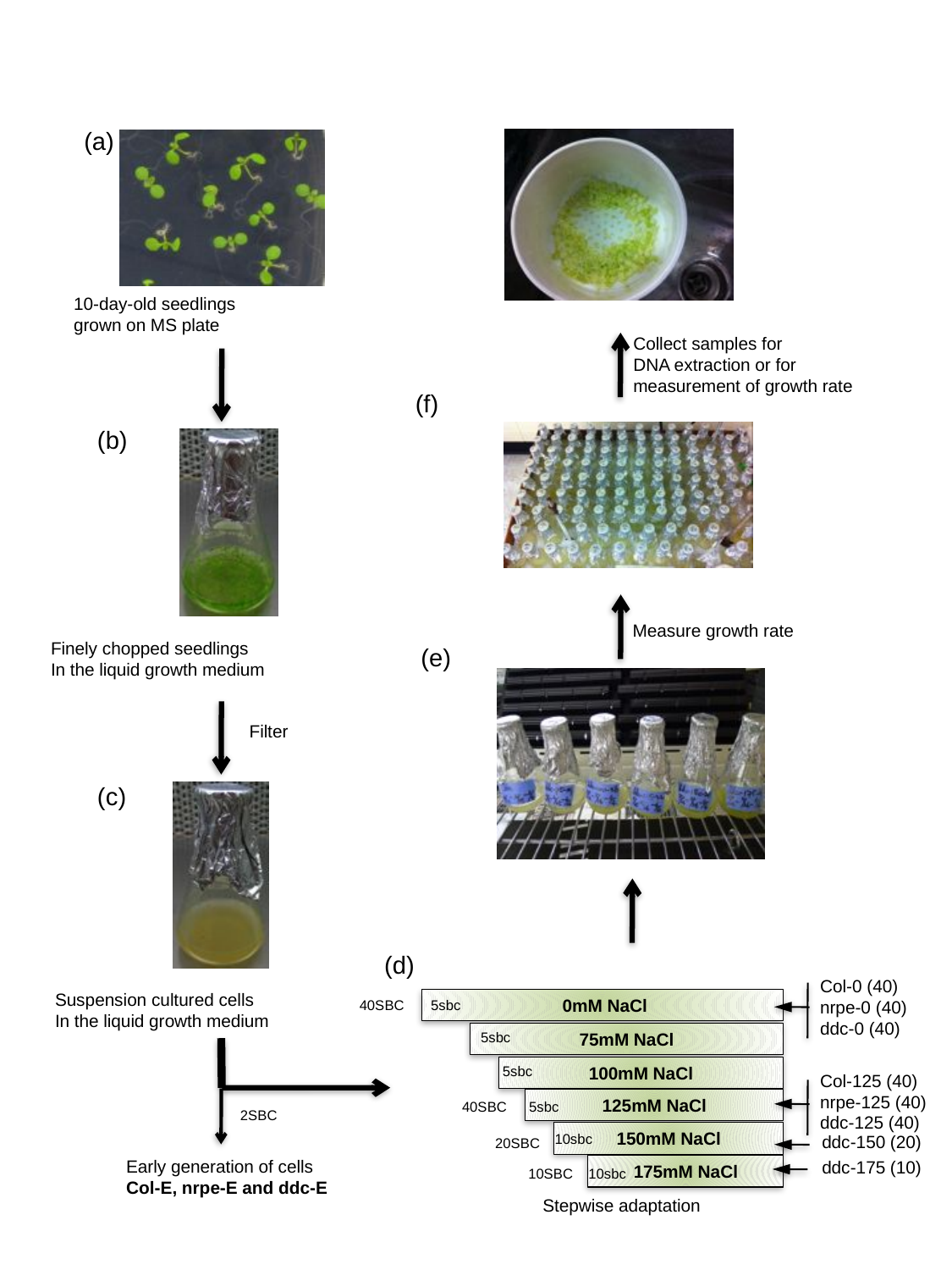

(a)
10-day-old seedlings
grown on MS plate
Collect samples for
DNA extraction or for
measurement of growth rate
(f)
(b)
Measure growth rate
Finely chopped seedlings
In the liquid growth medium
(e)
Filter
(c)
(d)
Col-0 (40)
nrpe-0 (40)
ddc-0 (40)
Suspension cultured cells
In the liquid growth medium
 0mM NaCl
40SBC
5sbc
5sbc
75mM NaCl
5sbc
100mM NaCl
Col-125 (40)
nrpe-125 (40)
ddc-125 (40)
125mM NaCl
40SBC
5sbc
2SBC
150mM NaCl
10sbc
ddc-150 (20)
20SBC
Early generation of cells
Col-E, nrpe-E and ddc-E
ddc-175 (10)
175mM NaCl
10SBC
10sbc
Stepwise adaptation

## Slide 2
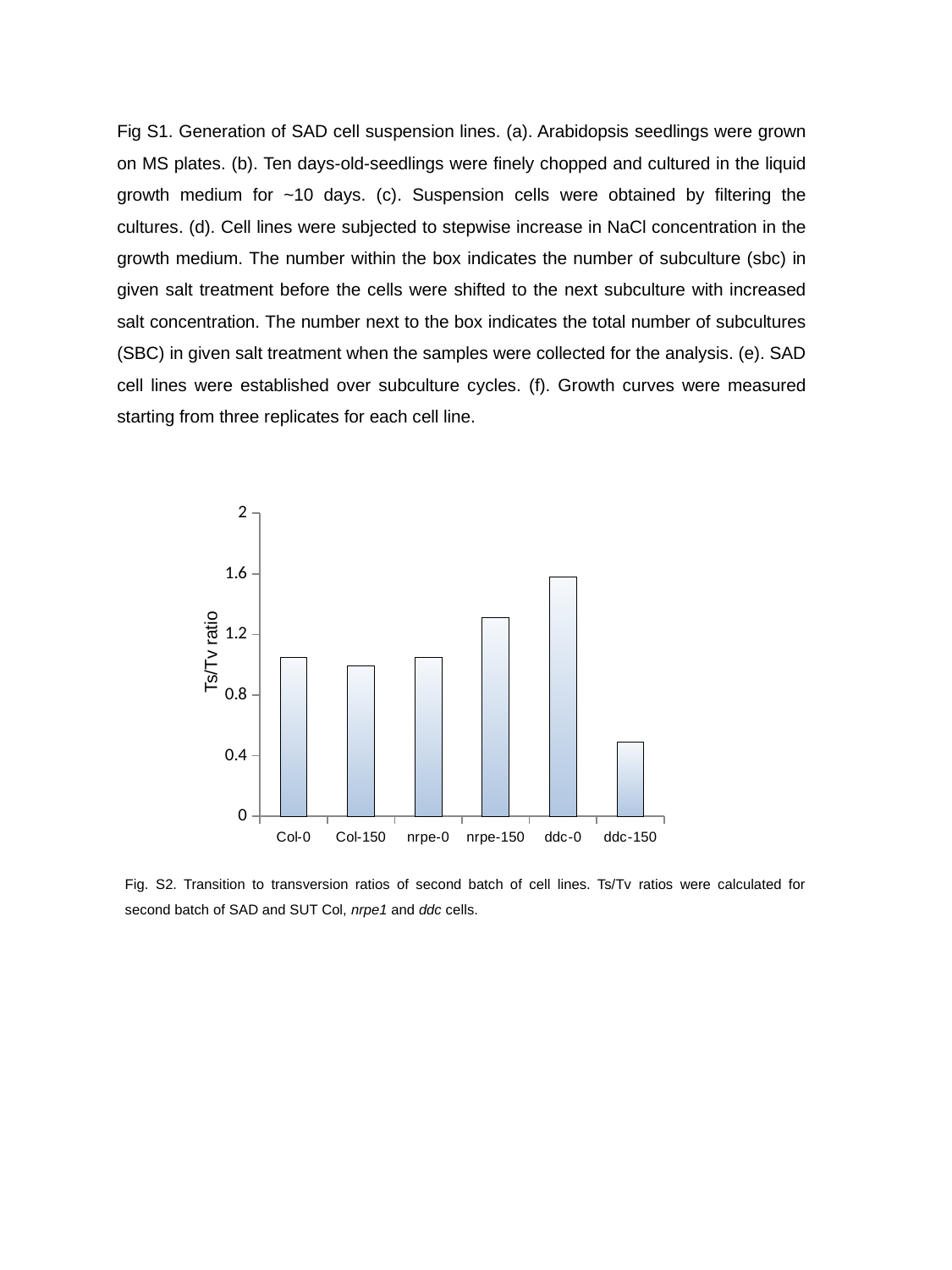

Fig S1. Generation of SAD cell suspension lines. (a). Arabidopsis seedlings were grown on MS plates. (b). Ten days-old-seedlings were finely chopped and cultured in the liquid growth medium for ~10 days. (c). Suspension cells were obtained by filtering the cultures. (d). Cell lines were subjected to stepwise increase in NaCl concentration in the growth medium. The number within the box indicates the number of subculture (sbc) in given salt treatment before the cells were shifted to the next subculture with increased salt concentration. The number next to the box indicates the total number of subcultures (SBC) in given salt treatment when the samples were collected for the analysis. (e). SAD cell lines were established over subculture cycles. (f). Growth curves were measured starting from three replicates for each cell line.
### Chart
| Category | |
|---|---|
| Col-0 | 1.047337959185846 |
| Col-150 | 0.995098241116022 |
| nrpe-0 | 1.049218269737793 |
| nrpe-150 | 1.312550371516636 |
| ddc-0 | 1.579747576781674 |
| ddc-150 | 0.491426939936196 |Ts/Tv ratio
Fig. S2. Transition to transversion ratios of second batch of cell lines. Ts/Tv ratios were calculated for second batch of SAD and SUT Col, nrpe1 and ddc cells.

## Slide 3
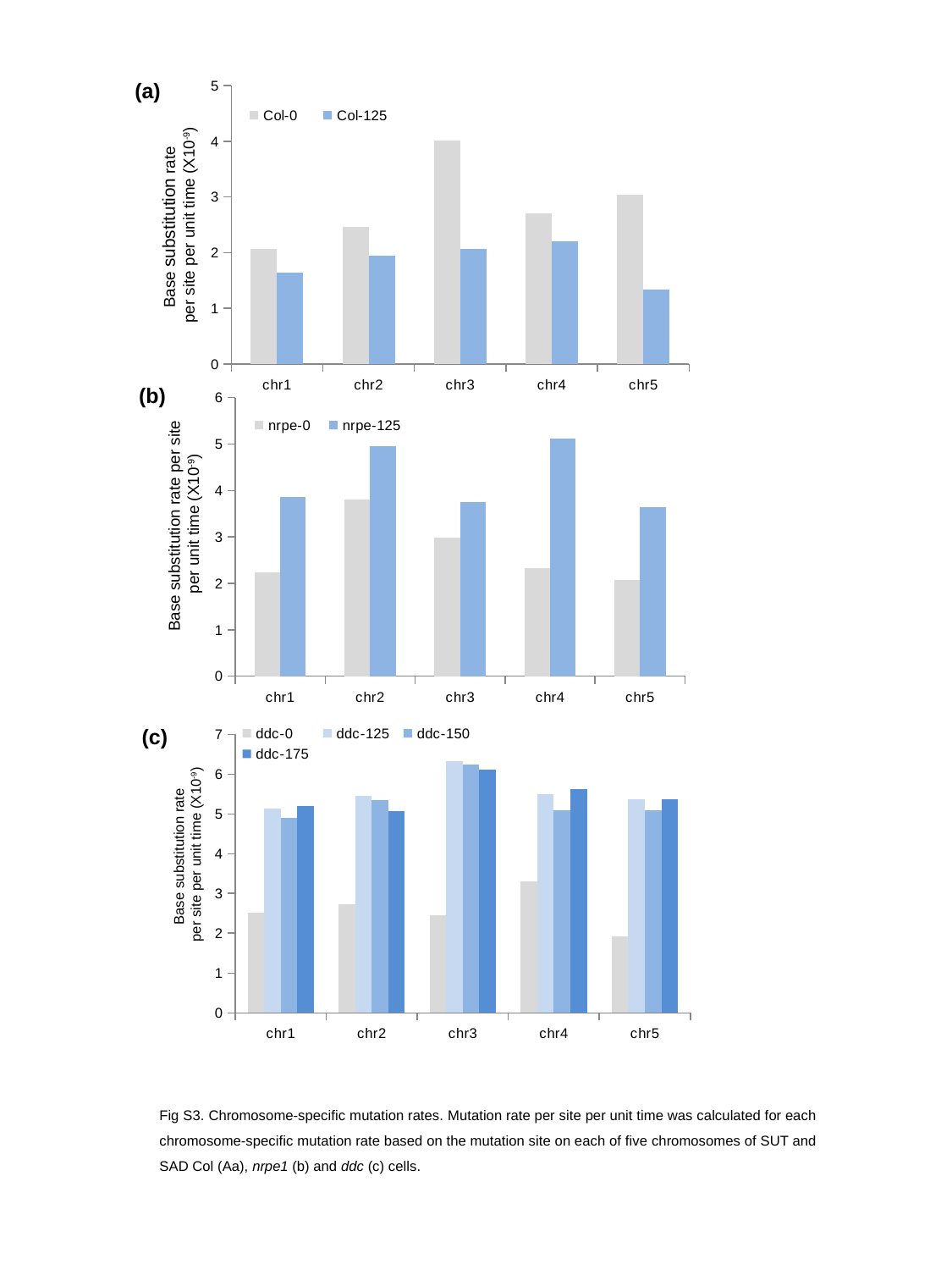

(a)
### Chart
| Category | Col-0 | Col-125 |
|---|---|---|
| chr1 | 2.069064636853009 | 1.64039520561534 |
| chr2 | 2.46090590604404 | 1.95204353128285 |
| chr3 | 4.01134030507131 | 2.074654135140717 |
| chr4 | 2.70609855469543 | 2.20137735800549 |
| chr5 | 3.042253733107569 | 1.34461896618975 |Base substitution rate
 per site per unit time (X10-9)
(b)
### Chart
| Category | nrpe-0 | nrpe-125 |
|---|---|---|
| chr1 | 2.23351562458883 | 3.866916629369518 |
| chr2 | 3.814558455440767 | 4.957715510930317 |
| chr3 | 2.98755077137142 | 3.746703928879 |
| chr4 | 2.31977080899113 | 5.115617127069447 |
| chr5 | 2.0724252788985 | 3.64140252769553 |Base substitution rate per site
 per unit time (X10-9)
(c)
### Chart
| Category | ddc-0 | ddc-125 | ddc-150 | ddc-175 |
|---|---|---|---|---|
| chr1 | 2.51835437951749 | 5.12680211964968 | 4.89235343553275 | 5.196881809383287 |
| chr2 | 2.71973910434846 | 5.46166996894838 | 5.33912100300738 | 5.067979965217937 |
| chr3 | 2.45868081518054 | 6.324265564293968 | 6.23678377920678 | 6.10915112088436 |
| chr4 | 3.30115179953512 | 5.49519640071802 | 5.087598317328717 | 5.632869269151297 |
| chr5 | 1.92293216326449 | 5.363463937465277 | 5.08637164816674 | 5.358172258398776 |Base substitution rate
 per site per unit time (X10-9)
Fig S3. Chromosome-specific mutation rates. Mutation rate per site per unit time was calculated for each chromosome-specific mutation rate based on the mutation site on each of five chromosomes of SUT and SAD Col (Aa), nrpe1 (b) and ddc (c) cells.

## Slide 4
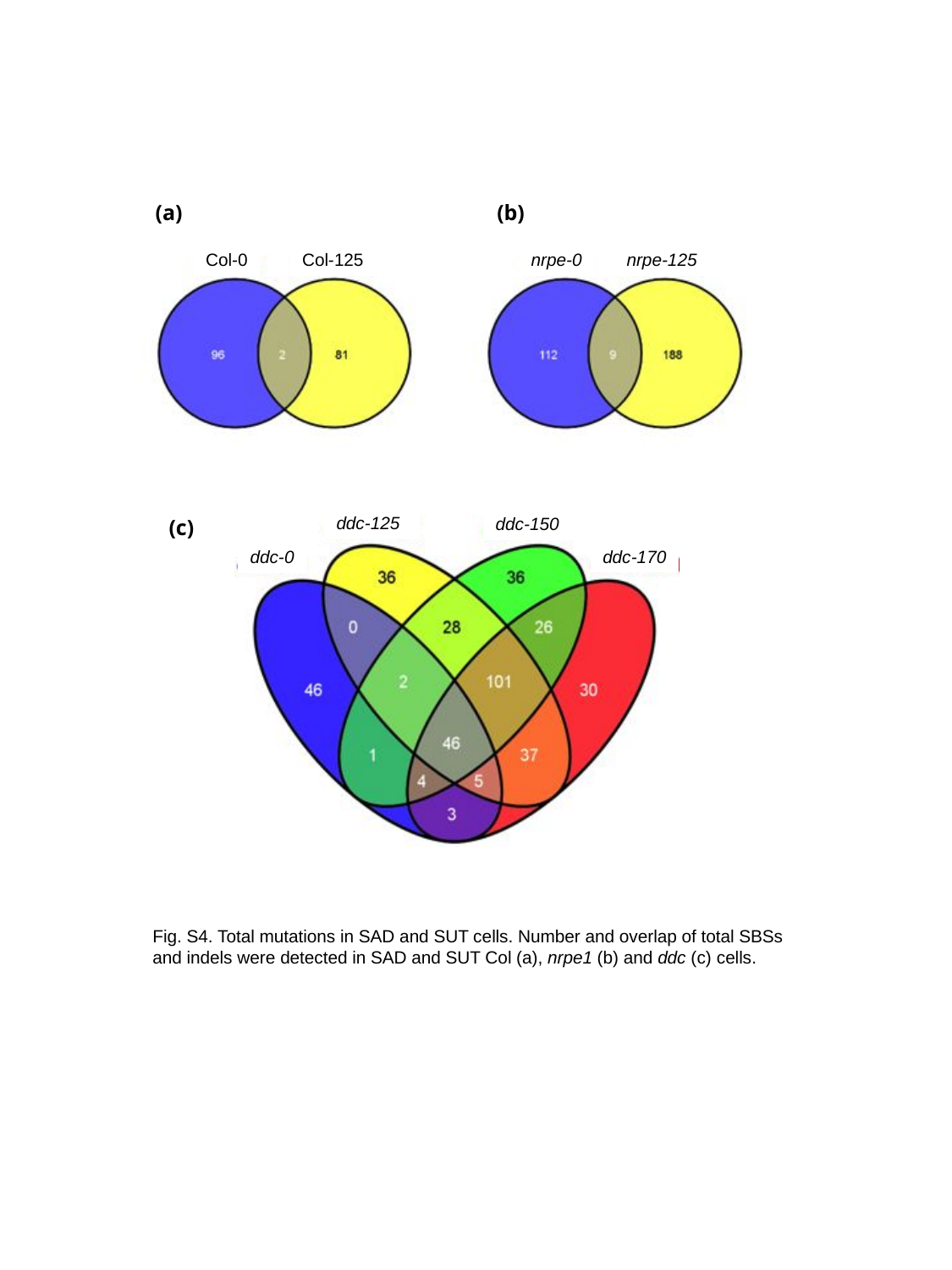

Col-0
Col-125
(a)
nrpe-0
nrpe-125
(b)
ddc-125
ddc-150
ddc-170
ddc-0
(c)
Fig. S4. Total mutations in SAD and SUT cells. Number and overlap of total SBSs and indels were detected in SAD and SUT Col (a), nrpe1 (b) and ddc (c) cells.
