# Supplementary material for: Non-CG DNA methylation-deficiency mutations enhance mutagenesis rates during salt adaptation in cultured Arabidopsis cells
Source: Stress Biol. 2021 Nov 15;1(1):12. doi: 10.1007/s44154-021-00013-2 (PMC10441993; doi:10.1007/s44154-021-00013-2)
Supplement: Supplementary file 2 — Additional file 2: Table S1. Number of mutations and their distributions among functional classes within the genome of 1st batch of SAD and SUT cells. Table S2. Relative proportion of deleterious nsSNPs in the first batch of SAD and SUT cells. Table S3. A list of total insertion and deletion (Indel) mutations in the first batch of SAD and SUT cells. Table S4. Number of mutations and their distributions among functional classes within the genome of 2nd batch of SAD and SUT cells. Table S5. A list of total coding region mutations in the first batch of SUT and SAD cells. [file 44154_2021_13_MOESM2_ESM.docx]

Table S1. Mapping efficiency of genomic DNA resequencing of cell lines

| 1^st^ batch Cell lines | Total Read | Mapped Read | Mapping efficiency | Coverage |
| --- | --- | --- | --- | --- |
| Col-E | 43,929,098 | 43,775,286 | 99.65 | 37.11 |
| *nrpe*-E | 41,337,319 | 41,197,084 | 99.66 | 34.92 |
| *ddc*-E | 45,632,479 | 45,458,243 | 99.62 | 38.53 |
| Col-0 | 25,510,346 | 25,344,061 | 99.35 | 21.48 |
| Col-125 | 40,465,841 | 40,299,488 | 99.59 | 34.16 |
| *nrpe*-0 | 30,999,647 | 30,919,904 | 99.74 | 26.21 |
| *nrpe*-125 | 65,160,359 | 65,065,935 | 99.86 | 55.16 |
| *ddc*-0 | 25,939,253 | 25,829,731 | 99.58 | 21.9 |
| *ddc*-125 | 47,134,799 | 46,966,029 | 99.64 | 39.81 |
| *ddc*-150 | 41,853,928 | 41,658,945 | 99.53 | 35.31 |
| *ddc*-175 | 47,332,309 | 47,151,959 | 99.62 | 39.97 |

| 2^nd^ batch Cell lines | Total Read | Mapped Read | Mapping efficiency | Coverage |
| --- | --- | --- | --- | --- |
| Col-0 | 25,476,812 | 25,416,913 | 99.76 | 21.55 |
| Col-150 | 25,312,846 | 25,257,593 | 99.78 | 21.41 |
| *nrpe*-0 | 21,023,146 | 20,967,995 | 99.74 | 17.77 |
| *nrpe*-150 | 25,687,935 | 25,625,027 | 99.76 | 21.22 |
| *ddc*-0 | 28,565,372 | 28,504,087 | 99.79 | 24.16 |
| *ddc*-150 | 21,000,033 | 20,933,108 | 99.68 | 17.74 |
|  |  |  |  |  |
|  |  |  |  |  |

Table S2. A list of total single base substitution (SBS) mutations in the first batch of

SAD and SUT cells

| Cell lines | Choromosome | Position | SBS | Region |
| --- | --- | --- | --- | --- |
| nrpe-0 | chr1 | 15191861 | T->C | TE |
| nrpe-0 | chr5 | 11709265 | A->T | IG |
| nrpe-0 | chr1 | 15695719 | C->T | TE |
| nrpe-0 | chr5 | 11505163 | T->A | TE |
| nrpe-0 | chr3 | 14792839 | C->A | TE |
| nrpe-0 | chr3 | 23457943 | C->T | Intron |
| nrpe-0 | chr3 | 23447463 | A->T | Coding |
| nrpe-0 | chr3 | 11820354 | C->T | pseudogene |
| nrpe-0 | chr5 | 16068302 | G->T | IG |
| nrpe-0 | chr1 | 22313366 | T->A | IG |
| nrpe-0 | chr2 | 19665474 | G->A | Coding |
| nrpe-0 | chr1 | 6884617 | G->C | IG |
| nrpe-0 | chr5 | 13456502 | T->A | IG |
| nrpe-0 | chr5 | 13881153 | C->A | IG |
| nrpe-0 | chr1 | 5298060 | T->A | ncRNA |
| nrpe-0 | chr5 | 4845773 | C->A | Intron |
| nrpe-0 | chr3 | 15715652 | G->A | TE |
| nrpe-0 | chr3 | 14986771 | C->G | TE |
| nrpe-0 | chr1 | 18915148 | C->T | UTR |
| nrpe-0 | chr5 | 8707212 | G->T | Coding |
| nrpe-0 | chr1 | 24008236 | A->T | Coding |
| nrpe-0 | chr1 | 7176153 | G->A | UTR |
| nrpe-0 | chr5 | 24539331 | T->C | IG |
| nrpe-0 | chr3 | 7899475 | C->A | Coding |
| nrpe-0 | chr4 | 3871961 | G->C | TE |
| nrpe-0 | chr2 | 3948702 | C->G | TE |
| nrpe-0 | chr1 | 4710643 | G->T | Coding |
| nrpe-0 | chr3 | 21679784 | T->A | IG |
| nrpe-0 | chr3 | 10169591 | C->A | Coding |
| nrpe-0 | chr2 | 1582821 | C->T | Coding |
| nrpe-0 | chr3 | 2107203 | T->C | Intron |
| nrpe-0 | chr2 | 8931620 | G->C | ncRNA |
| nrpe-0 | chr1 | 30087386 | G->A | Intron |
| nrpe-0 | chr1 | 10626112 | A->G | Coding |
| nrpe-0 | chr4 | 3634172 | A->C | TE |
| nrpe-0 | chr5 | 12456018 | G->T | TE |
| nrpe-0 | chr1 | 21210302 | C->A | TE |
| nrpe-0 | chr4 | 18304921 | T->C | ncRNA |
| nrpe-0 | chr4 | 16397285 | A->T | TE |
| nrpe-0 | chr5 | 5674618 | G->A | IG |
| nrpe-0 | chr2 | 15298169 | A->C | Intron |
| nrpe-0 | chr2 | 3923520 | A->T | TE |
| nrpe-0 | chr1 | 1337102 | T->C | Coding |
| nrpe-0 | chr5 | 21931433 | G->T | TE |
| nrpe-0 | chr1 | 19516953 | A->G | Coding |
| nrpe-0 | chr3 | 13589731 | G->C | IG |
| nrpe-0 | chr4 | 6296599 | T->C | Coding |
| nrpe-0 | chr4 | 3981051 | A->C | IG |
| nrpe-0 | chr5 | 10816703 | G->A | TE |
| nrpe-0 | chr3 | 21288131 | A->T | IG |
| nrpe-0 | chr3 | 13587066 | C->T | IG |
| nrpe-0 | chr3 | 12247101 | A->G | TE |
| nrpe-0 | chr1 | 15097537 | C->A | TE |
| nrpe-0 | chr3 | 3874836 | T->G | Coding |
| nrpe-0 | chr2 | 4331112 | A->G | IG |
| nrpe-0 | chr2 | 2042257 | G->A | TE |
| nrpe-0 | chr3 | 17926839 | A->G | Intron |
| nrpe-0 | chr5 | 20795975 | G->A | ncRNA |
| nrpe-0 | chr2 | 3468902 | T->C | IG |
| nrpe-0 | chr5 | 13451291 | A->T | IG |
| nrpe-0 | chr1 | 13350476 | C->T | IG |
| nrpe-0 | chr2 | 3367209 | G->A | IG |
| nrpe-0 | chr3 | 8402215 | A->G | IG |
| nrpe-0 | chr3 | 13832875 | T->A | TE |
| nrpe-0 | chr2 | 15261490 | G->A | Coding |
| nrpe-0 | chr4 | 13333220 | A->G | IG |
| nrpe-0 | chr2 | 3308822 | T->A | TE |
| nrpe-0 | chr5 | 4563822 | G->A | IG |
| nrpe-0 | chr2 | 5695188 | G->A | Coding |
| nrpe-0 | chr5 | 21992085 | C->A | Coding |
| nrpe-0 | chr4 | 13832028 | C->A | Coding |
| nrpe-0 | chr2 | 13460146 | G->C | Coding |
| nrpe-0 | chr2 | 4936374 | T->A | TE |
| nrpe-0 | chr1 | 9486795 | G->A | IG |
| nrpe-0 | chr3 | 8979513 | A->G | ncRNA |
| nrpe-0 | chr2 | 528552 | A->G | Intron |
| nrpe-0 | chr4 | 1122814 | C->G | IG |
| nrpe-0 | chr2 | 19455645 | G->T | Coding |
| nrpe-0 | chr5 | 15736467 | C->A | Coding |
| nrpe-0 | chr2 | 3308797 | C->A | TE |
| nrpe-0 | chr4 | 3841598 | A->T | TE |
| nrpe-0 | chr2 | 60486 | G->A | TE |
| nrpe-0 | chr1 | 17571287 | C->T | IG |
| nrpe-0 | chr4 | 5396244 | G->C | IG |
| nrpe-0 | chr4 | 3277480 | C->A | TE |
| nrpe-0 | chr3 | 3113880 | G->T | TE |
| nrpe-0 | chr5 | 11736541 | T->C | IG |
| nrpe-0 | chr2 | 1650999 | G->C | TE |
| nrpe-0 | chr1 | 10475076 | G->T | UTR |
| nrpe-0 | chr5 | 11185379 | G->C | TE |
| nrpe-0 | chr4 | 3981315 | T->A | IG |
| nrpe-0 | chr2 | 6845658 | G->T | TE |
| nrpe-0 | chr3 | 3219203 | T->C | UTR |
| nrpe-0 | chr4 | 3988625 | C->T | TE |
| nrpe-0 | chr3 | 15715591 | C->T | TE |
| nrpe-0 | chr3 | 14220189 | T->A | IG |
| nrpe-0 | chr2 | 5999384 | T->A | TE |
| nrpe-0 | chr4 | 4434077 | T->C | TE |
| nrpe-0 | chr2 | 2570128 | C->A | TE |
| nrpe-0 | chr1 | 16933515 | G->T | IG |
| nrpe-0 | chr2 | 17396401 | A->T | IG |
| nrpe-0 | chr2 | 3322401 | T->C | IG |
| nrpe-0 | chr2 | 6943956 | A->G | TE |
| nrpe-0 | chr1 | 24760122 | A->T | IG |
| nrpe-0 | chr1 | 18428055 | G->T | IG |
| nrpe-0 | chr4 | 3903451 | A->G | TE |
| nrpe-0 | chr3 | 23427211 | T->G | Coding |
| nrpe-0 | chr3 | 23088142 | T->G | Intron |
| nrpe-0 | chr1 | 29096518 | G->T | Coding |
| nrpe-0 | chr5 | 683244 | T->C | Coding |
| nrpe-0 | chr5 | 724241 | G->C | IG |
| nrpe-0 | chr2 | 1634955 | G->A | TE |
| nrpe-0 | chr1 | 28649824 | C->T | Coding |
| nrpe-0 | chr1 | 22425018 | T->A | Intron |
| nrpe-0 | chr2 | 5199506 | T->C | TE |
| nrpe-0 | chr4 | 3988921 | G->A | TE |
| nrpe-0 | chr5 | 19022136 | C->A | IG |
| nrpe-0 | chr3 | 21559104 | T->A | ncRNA |
| nrpe-0 | chr1 | 11009933 | G->T | Coding |
| nrpe-0 | chr3 | 9989139 | T->C | TE |
| nrpe-0 | chr2 | 9560400 | C->A | IG |
| ddc-0 | chr4 | 4354852 | C->T | IG |
| ddc-0 | chr3 | 13806841 | G->A | TE |
| ddc-0 | chr1 | 15169786 | T->G | TE |
| ddc-0 | chr1 | 14721422 | C->G | IG |
| ddc-0 | chr1 | 2394372 | A->C | IG |
| ddc-0 | chr1 | 15093489 | C->T | TE |
| ddc-0 | chr5 | 12010304 | G->A | TE |
| ddc-0 | chr4 | 1794708 | C->T | TE |
| ddc-0 | chr2 | 3085452 | A->G | TE |
| ddc-0 | chr1 | 19327483 | A->T | TE |
| ddc-0 | chr5 | 11810674 | A->T | TE |
| ddc-0 | chr3 | 12153243 | A->T | TE |
| ddc-0 | chr2 | 3951070 | A->G | TE |
| ddc-0 | chr3 | 11172241 | C->T | TE |
| ddc-0 | chr3 | 13945274 | A->T | TE |
| ddc-0 | chr4 | 13719900 | C->T | Coding |
| ddc-0 | chr5 | 25779888 | T->C | Coding |
| ddc-0 | chr5 | 15044539 | C->A | IG |
| ddc-0 | chr4 | 11518408 | C->T | Coding |
| ddc-0 | chr2 | 6077383 | T->C | TE |
| ddc-0 | chr1 | 11031812 | G->T | IG |
| ddc-0 | chr5 | 7685200 | T->C | IG |
| ddc-0 | chr1 | 694908 | T->C | Coding |
| ddc-0 | chr4 | 13545254 | A->G | Coding |
| ddc-0 | chr1 | 22111234 | A->G | IG |
| ddc-0 | chr5 | 11216737 | G->C | TE |
| ddc-0 | chr3 | 16936238 | T->A | Coding |
| ddc-0 | chr2 | 15416087 | A->C | Coding |
| ddc-0 | chr2 | 1173937 | A->G | Coding |
| ddc-0 | chr5 | 15673730 | G->A | TE |
| ddc-0 | chr2 | 11397684 | T->G | TE |
| ddc-0 | chr5 | 12039538 | A->G | TE |
| ddc-0 | chr2 | 2797886 | G->A | TE |
| ddc-0 | chr1 | 13872333 | G->T | IG |
| ddc-0 | chr4 | 3963801 | T->C | TE |
| ddc-0 | chr1 | 15085549 | G->T | Intron |
| ddc-0 | chr5 | 22020824 | A->T | TE |
| ddc-0 | chr4 | 3874729 | C->T | TE |
| ddc-0 | chr4 | 3850838 | G->A | TE |
| ddc-0 | chr1 | 24023720 | G->A | UTR |
| ddc-0 | chr2 | 3624168 | G->A | Intron |
| ddc-0 | chr5 | 20741722 | A->T | IG |
| ddc-0 | chr3 | 12252331 | C->G | TE |
| ddc-0 | chr5 | 9255065 | C->G | UTR |
| ddc-0 | chr3 | 17337790 | G->T | IG |
| ddc-0 | chr3 | 11876405 | G->A | TE |
| ddc-0 | chr1 | 19778712 | C->T | Coding |
| ddc-0 | chr5 | 22020813 | A->T | TE |
| ddc-0 | chr4 | 17774 | A->G | TE |
| ddc-0 | chr1 | 2452718 | A->C | Coding |
| ddc-0 | chr2 | 11431724 | C->A | Coding |
| ddc-0 | chr2 | 6374206 | C->T | TE |
| ddc-0 | chr3 | 14210783 | T->C | IG |
| ddc-0 | chr5 | 12128752 | G->A | TE |
| ddc-0 | chr2 | 3965964 | A->G | TE |
| ddc-0 | chr3 | 651702 | T->C | Intron |
| ddc-0 | chr1 | 15446122 | C->T | TE |
| ddc-0 | chr2 | 46187 | G->A | TE |
| ddc-0 | chr5 | 592036 | C->T | Coding |
| ddc-0 | chr4 | 3055786 | T->G | TE |
| ddc-0 | chr1 | 9090698 | T->A | Coding |
| ddc-0 | chr1 | 14931185 | G->T | IG |
| ddc-0 | chr4 | 15909750 | T->A | Intron |
| ddc-0 | chr4 | 2100272 | G->C | IG |
| ddc-0 | chr4 | 5314361 | C->A | TE |
| ddc-0 | chr1 | 20853749 | T->C | Intron |
| ddc-0 | chr1 | 10090435 | G->T | ncRNA |
| ddc-0 | chr1 | 14094300 | A->G | TE |
| ddc-0 | chr5 | 23322292 | C->T | Coding |
| ddc-0 | chr1 | 15952190 | T->A | TE |
| ddc-0 | chr2 | 21935 | G->A | TE |
| ddc-0 | chr5 | 9509365 | C->A | Coding |
| ddc-0 | chr1 | 26513289 | C->A | Coding |
| ddc-0 | chr2 | 357989 | T->C | Coding |
| ddc-0 | chr4 | 4299762 | T->A | TE |
| ddc-0 | chr1 | 9267492 | A->G | IG |
| ddc-0 | chr5 | 11723283 | T->A | IG |
| ddc-0 | chr3 | 5326497 | T->A | Intron |
| ddc-0 | chr3 | 12698777 | C->T | TE |
| ddc-0 | chr3 | 13709142 | G->A | IG |
| ddc-0 | chr2 | 818647 | A->T | IG |
| ddc-0 | chr3 | 16668788 | C->A | Intron |
| ddc-0 | chr5 | 11718856 | G->A | TE |
| ddc-0 | chr3 | 12869548 | T->A | TE |
| ddc-0 | chr1 | 20534312 | C->G | pseudogene |
| ddc-0 | chr2 | 17345248 | T->A | Intron |
| ddc-0 | chr1 | 16519831 | A->C | TE |
| ddc-0 | chr4 | 2744311 | C->G | Coding |
| ddc-0 | chr1 | 16628678 | C->T | TE |
| ddc-0 | chr3 | 20465697 | G->C | Coding |
| ddc-0 | chr2 | 17090800 | A->C | TE |
| ddc-0 | chr4 | 7819572 | T->A | TE |
| ddc-0 | chr3 | 12900506 | C->T | TE |
| ddc-0 | chr4 | 15085587 | C->T | Intron |
| ddc-0 | chr3 | 20813597 | A->G | IG |
| ddc-0 | chr4 | 11473217 | G->T | IG |
| ddc-0 | chr4 | 4032413 | G->A | TE |
| ddc-0 | chr3 | 10141638 | A->G | Intron |
| ddc-0 | chr4 | 3700916 | T->A | TE |
| ddc-0 | chr4 | 3002960 | C->G | TE |
| ddc-0 | chr2 | 2031495 | G->T | TE |
| ddc-0 | chr3 | 14683901 | G->A | TE |
| ddc-0 | chr1 | 24024976 | G->A | Intron |
| ddc-0 | chr1 | 15098291 | A->G | TE |
| ddc-0 | chr3 | 13229391 | C->T | TE |
| ddc-0 | chr4 | 3980616 | C->G | IG |
| ddc-0 | chr4 | 3995564 | C->T | TE |
| Col-0 | chr3 | 14224663 | A->G | IG |
| Col-0 | chr3 | 12207407 | G->T | TE |
| Col-0 | chr5 | 11956614 | T->A | IG |
| Col-0 | chr3 | 13587017 | C->G | IG |
| Col-0 | chr1 | 18902046 | C->G | Coding |
| Col-0 | chr3 | 14081415 | C->T | TE |
| Col-0 | chr2 | 6360816 | C->G | Coding |
| Col-0 | chr4 | 9507041 | A->G | ncRNA |
| Col-0 | chr5 | 18677662 | G->A | Intron |
| Col-0 | chr5 | 12640660 | C->T | TE |
| Col-0 | chr1 | 15159192 | C->A | IG |
| Col-0 | chr3 | 17872442 | G->A | IG |
| Col-0 | chr3 | 14176876 | G->T | TE |
| Col-0 | chr5 | 12365089 | C->G | pseudogene |
| Col-0 | chr2 | 3319908 | A->T | IG |
| Col-0 | chr3 | 3978111 | C->G | Intron |
| Col-0 | chr1 | 29869381 | G->A | Coding |
| Col-0 | chr4 | 552019 | C->G | Coding |
| Col-0 | chr4 | 9350151 | C->A | Coding |
| Col-0 | chr1 | 16508446 | C->T | IG |
| Col-0 | chr2 | 21935 | G->A | TE |
| Col-0 | chr5 | 14327593 | A->G | IG |
| Col-0 | chr5 | 12436234 | A->T | TE |
| Col-0 | chr5 | 10947012 | G->A | TE |
| Col-0 | chr3 | 17373019 | C->A | Coding |
| Col-0 | chr1 | 19082591 | T->G | IG |
| Col-0 | chr3 | 21775172 | A->C | IG |
| Col-0 | chr4 | 4058163 | A->G | IG |
| Col-0 | chr3 | 13589030 | G->C | IG |
| Col-0 | chr2 | 4521864 | G->A | TE |
| Col-0 | chr3 | 3481330 | T->G | UTR |
| Col-0 | chr3 | 18049742 | T->C | Coding |
| Col-0 | chr3 | 13587070 | A->C | IG |
| Col-0 | chr1 | 16586500 | G->A | TE |
| Col-0 | chr1 | 19498455 | C->A | ncRNA |
| Col-0 | chr4 | 7048728 | C->G | Coding |
| Col-0 | chr3 | 16561341 | A->C | TE |
| Col-0 | chr3 | 16250890 | A->G | IG |
| Col-0 | chr3 | 13776912 | T->C | IG |
| Col-0 | chr5 | 12046854 | G->C | IG |
| Col-0 | chr5 | 11724856 | C->G | IG |
| Col-0 | chr3 | 16136536 | G->A | TE |
| Col-0 | chr4 | 9660508 | G->C | IG |
| Col-0 | chr3 | 14222611 | G->A | IG |
| Col-0 | chr5 | 12017617 | G->T | TE |
| Col-0 | chr4 | 2953555 | G->C | IG |
| Col-0 | chr2 | 4674401 | G->A | TE |
| Col-0 | chr5 | 15886843 | C->T | TE |
| Col-0 | chr4 | 3537440 | C->A | TE |
| Col-0 | chr1 | 27486085 | A->G | Coding |
| Col-0 | chr3 | 12472061 | G->A | IG |
| Col-0 | chr5 | 11051192 | A->T | TE |
| Col-0 | chr1 | 20091032 | T->A | IG |
| Col-0 | chr3 | 13767788 | G->A | IG |
| Col-0 | chr5 | 11684037 | C->T | IG |
| Col-0 | chr1 | 13651606 | C->T | TE |
| Col-0 | chr4 | 4749573 | C->T | TE |
| Col-0 | chr1 | 15203167 | T->A | TE |
| Col-0 | chr1 | 21345660 | T->C | Coding |
| Col-0 | chr3 | 5126043 | G->T | IG |
| Col-0 | chr2 | 3319818 | C->A | IG |
| Col-0 | chr2 | 16112922 | T->C | IG |
| Col-0 | chr3 | 13778739 | C->T | IG |
| Col-0 | chr5 | 780775 | C->T | IG |
| Col-0 | chr3 | 11182947 | T->C | TE |
| Col-0 | chr5 | 12776793 | G->A | TE |
| Col-0 | chr3 | 15254593 | G->A | TE |
| Col-0 | chr4 | 3181521 | C->T | TE |
| Col-0 | chr3 | 13776134 | C->T | IG |
| Col-0 | chr3 | 15305374 | C->T | TE |
| Col-0 | chr1 | 18160295 | C->T | TE |
| Col-0 | chr4 | 3695567 | A->G | TE |
| Col-0 | chr4 | 12443191 | G->A | Coding |
| Col-0 | chr4 | 3144081 | G->A | TE |
| Col-0 | chr5 | 11321553 | T->C | TE |
| Col-0 | chr1 | 16527315 | C->T | TE |
| Col-0 | chr1 | 26884425 | C->A | Coding |
| Col-0 | chr4 | 3673468 | C->T | TE |
| Col-0 | chr5 | 13783915 | G->T | TE |
| Col-0 | chr1 | 11323292 | G->A | Coding |
| Col-0 | chr4 | 3058204 | C->T | IG |
| Col-0 | chr5 | 17913171 | T->C | IG |
| Col-0 | chr2 | 636041 | C->T | pseudogene |
| Col-0 | chr1 | 25038040 | A->T | Intron |
| Col-0 | chr4 | 1724739 | G->A | IG |
| Col-0 | chr2 | 6950141 | C->T | UTR |
| Col-0 | chr2 | 6549544 | A->C | TE |
| Col-0 | chr2 | 1832065 | T->G | TE |
| Col-0 | chr5 | 12640768 | C->G | TE |
| Col-0 | chr2 | 3210555 | A->G | TE |
| Col-0 | chr1 | 15330268 | T->A | TE |
| Col-0 | chr2 | 6820583 | C->G | TE |
| Col-0 | chr2 | 3617567 | T->G | Intron |
| Col-0 | chr3 | 17699129 | C->G | Intron |
| Col-0 | chr1 | 15090637 | C->A | TE |
| Col-0 | chr5 | 11777802 | C->A | TE |
| Col-0 | chr1 | 13483702 | A->G | TE |
| Col-0 | chr2 | 7994014 | A->T | Intron |
| Col-125 | chr4 | 3590556 | A->G | TE |
| Col-125 | chr3 | 5895434 | C->T | IG |
| Col-125 | chr5 | 3567328 | T->C | Intron |
| Col-125 | chr2 | 5199534 | G->T | TE |
| Col-125 | chr1 | 19246203 | G->C | TE |
| Col-125 | chr3 | 15257635 | C->T | TE |
| Col-125 | chr3 | 13778739 | C->T | IG |
| Col-125 | chr5 | 18875085 | G->C | IG |
| Col-125 | chr4 | 3462177 | C->T | TE |
| Col-125 | chr1 | 18567608 | T->C | pseudogene |
| Col-125 | chr1 | 17485278 | C->A | Coding |
| Col-125 | chr5 | 22384446 | A->T | Intron |
| Col-125 | chr1 | 11049492 | G->A | TE |
| Col-125 | chr1 | 25742499 | A->G | IG |
| Col-125 | chr1 | 26623662 | A->T | Coding |
| Col-125 | chr2 | 5565387 | C->T | TE |
| Col-125 | chr5 | 13257864 | A->G | TE |
| Col-125 | chr1 | 9002920 | C->A | Intron |
| Col-125 | chr3 | 15324044 | G->A | TE |
| Col-125 | chr3 | 14113695 | C->T | TE |
| Col-125 | chr4 | 1715204 | T->A | IG |
| Col-125 | chr2 | 2488473 | C->G | TE |
| Col-125 | chr5 | 25154867 | G->T | Intron |
| Col-125 | chr2 | 19108785 | C->T | IG |
| Col-125 | chr2 | 11568166 | A->G | Coding |
| Col-125 | chr1 | 4356929 | A->G | Coding |
| Col-125 | chr4 | 1122814 | C->G | IG |
| Col-125 | chr1 | 13973476 | G->A | TE |
| Col-125 | chr2 | 9608847 | C->G | Intron |
| Col-125 | chr5 | 18193695 | T->G | TE |
| Col-125 | chr1 | 5393436 | G->A | Coding |
| Col-125 | chr5 | 11431807 | T->G | TE |
| Col-125 | chr5 | 11330938 | G->C | TE |
| Col-125 | chr4 | 9260099 | G->A | UTR |
| Col-125 | chr2 | 352086 | G->A | TE |
| Col-125 | chr3 | 18392710 | C->T | IG |
| Col-125 | chr2 | 2543613 | T->A | TE |
| Col-125 | chr4 | 4391646 | C->A | TE |
| Col-125 | chr1 | 16524164 | G->A | TE |
| Col-125 | chr1 | 15828159 | G->T | TE |
| Col-125 | chr5 | 19755887 | C->T | IG |
| Col-125 | chr3 | 6838756 | G->T | TE |
| Col-125 | chr2 | 1630554 | C->G | TE |
| Col-125 | chr2 | 673251 | A->G | IG |
| Col-125 | chr1 | 23930094 | C->G | TE |
| Col-125 | chr4 | 3281068 | C->G | TE |
| Col-125 | chr3 | 14435462 | G->T | TE |
| Col-125 | chr2 | 14924749 | C->T | Intron |
| Col-125 | chr5 | 19494051 | G->A | TE |
| Col-125 | chr1 | 9260067 | G->A | Coding |
| Col-125 | chr4 | 3135214 | C->G | TE |
| Col-125 | chr5 | 7595451 | T->A | Intron |
| Col-125 | chr2 | 1812172 | C->T | TE |
| Col-125 | chr4 | 3614781 | C->A | TE |
| Col-125 | chr4 | 6024495 | A->G | TE |
| Col-125 | chr4 | 5070904 | G->T | TE |
| Col-125 | chr1 | 15084101 | C->A | Coding |
| Col-125 | chr2 | 3367552 | C->T | IG |
| Col-125 | chr3 | 8851387 | T->A | TE |
| Col-125 | chr4 | 12310807 | C->T | Coding |
| Col-125 | chr1 | 13153797 | A->T | Coding |
| Col-125 | chr5 | 5735655 | T->C | Coding |
| Col-125 | chr3 | 3428978 | G->C | IG |
| Col-125 | chr3 | 15258868 | G->A | TE |
| Col-125 | chr4 | 1623241 | G->A | TE |
| Col-125 | chr2 | 2295661 | C->T | IG |
| Col-125 | chr4 | 13426135 | T->C | Coding |
| Col-125 | chr2 | 15655568 | A->G | Intron |
| Col-125 | chr3 | 2094498 | C->G | IG |
| Col-125 | chr3 | 16713906 | A->C | TE |
| Col-125 | chr3 | 20407144 | T->C | IG |
| Col-125 | chr1 | 9688868 | T->A | TE |
| Col-125 | chr3 | 13767788 | G->A | IG |
| Col-125 | chr4 | 11784206 | T->A | Intron |
| Col-125 | chr1 | 5444024 | C->T | IG |
| Col-125 | chr1 | 24956655 | G->A | IG |
| Col-125 | chr3 | 15715652 | G->A | TE |
| Col-125 | chr4 | 4365616 | G->T | TE |
| Col-125 | chr3 | 13280407 | C->A | TE |
| Col-125 | chr3 | 21547112 | T->A | IG |
| Col-125 | chr5 | 8051304 | C->A | IG |
| Col-125 | chr5 | 12010255 | G->T | TE |
| Col-125 | chr3 | 20540810 | G->A | TE |
| nrpe-125 | chr4 | 3871961 | G->C | TE |
| nrpe-125 | chr4 | 1337072 | T->G | Coding |
| nrpe-125 | chr5 | 9471162 | A->G | TE |
| nrpe-125 | chr5 | 10887644 | G->T | TE |
| nrpe-125 | chr3 | 1940729 | G->T | IG |
| nrpe-125 | chr2 | 15958542 | T->C | IG |
| nrpe-125 | chr2 | 4194418 | G->A | TE |
| nrpe-125 | chr1 | 16318469 | T->C | Coding |
| nrpe-125 | chr5 | 14464821 | G->T | Coding |
| nrpe-125 | chr4 | 4249117 | A->G | TE |
| nrpe-125 | chr4 | 1505639 | T->C | UTR |
| nrpe-125 | chr4 | 1304155 | C->T | IG |
| nrpe-125 | chr3 | 20432501 | G->A | IG |
| nrpe-125 | chr2 | 5369101 | G->A | TE |
| nrpe-125 | chr1 | 14045175 | A->G | TE |
| nrpe-125 | chr2 | 6845658 | G->T | TE |
| nrpe-125 | chr2 | 3319818 | C->A | IG |
| nrpe-125 | chr1 | 17851921 | T->C | Intron |
| nrpe-125 | chr5 | 24948003 | A->G | Intron |
| nrpe-125 | chr3 | 22747029 | G->T | Coding |
| nrpe-125 | chr1 | 23170066 | A->C | Intron |
| nrpe-125 | chr1 | 20387501 | G->A | UTR |
| nrpe-125 | chr5 | 13251595 | T->C | TE |
| nrpe-125 | chr3 | 14809345 | A->G | TE |
| nrpe-125 | chr4 | 5955302 | G->C | TE |
| nrpe-125 | chr4 | 16958959 | G->T | Coding |
| nrpe-125 | chr2 | 4043193 | G->C | TE |
| nrpe-125 | chr2 | 3228143 | C->T | TE |
| nrpe-125 | chr2 | 60486 | G->A | TE |
| nrpe-125 | chr5 | 23387738 | G->T | Coding |
| nrpe-125 | chr4 | 1375409 | G->T | Intron |
| nrpe-125 | chr5 | 11667415 | G->C | IG |
| nrpe-125 | chr1 | 23179733 | G->A | Coding |
| nrpe-125 | chr5 | 7279557 | G->A | Intron |
| nrpe-125 | chr4 | 6416383 | C->A | Coding |
| nrpe-125 | chr3 | 10726911 | A->T | pseudogene |
| nrpe-125 | chr1 | 22368717 | T->G | IG |
| nrpe-125 | chr2 | 3584360 | T->C | IG |
| nrpe-125 | chr4 | 18526856 | G->A | Coding |
| nrpe-125 | chr1 | 23327880 | C->T | TE |
| nrpe-125 | chr5 | 22146723 | C->T | UTR |
| nrpe-125 | chr4 | 1338602 | T->A | IG |
| nrpe-125 | chr1 | 10698068 | C->T | TE |
| nrpe-125 | chr5 | 18624163 | A->G | Intron |
| nrpe-125 | chr3 | 11420608 | G->C | TE |
| nrpe-125 | chr3 | 7167375 | C->T | Coding |
| nrpe-125 | chr2 | 47430 | C->T | TE |
| nrpe-125 | chr2 | 11968742 | T->A | IG |
| nrpe-125 | chr2 | 3549013 | C->T | TE |
| nrpe-125 | chr3 | 6659222 | C->A | Coding |
| nrpe-125 | chr1 | 17136192 | G->C | pseudogene |
| nrpe-125 | chr1 | 22177247 | C->A | Coding |
| nrpe-125 | chr3 | 3187093 | A->G | Coding |
| nrpe-125 | chr2 | 3902285 | T->A | IG |
| nrpe-125 | chr2 | 3797764 | T->A | IG |
| nrpe-125 | chr1 | 15104950 | T->A | TE |
| nrpe-125 | chr1 | 25316785 | G->A | Coding |
| nrpe-125 | chr1 | 30062162 | T->G | Coding |
| nrpe-125 | chr2 | 3020173 | T->C | TE |
| nrpe-125 | chr5 | 12918894 | T->G | IG |
| nrpe-125 | chr3 | 19265520 | G->A | Coding |
| nrpe-125 | chr4 | 11099884 | C->T | Coding |
| nrpe-125 | chr5 | 11255307 | T->C | IG |
| nrpe-125 | chr2 | 2475475 | C->T | IG |
| nrpe-125 | chr4 | 1367481 | C->A | Coding |
| nrpe-125 | chr5 | 3075778 | C->T | Coding |
| nrpe-125 | chr4 | 4992419 | A->G | IG |
| nrpe-125 | chr5 | 7725805 | G->T | IG |
| nrpe-125 | chr1 | 13771212 | G->A | TE |
| nrpe-125 | chr5 | 12042267 | C->T | TE |
| nrpe-125 | chr3 | 17447392 | A->G | Intron |
| nrpe-125 | chr3 | 12247101 | A->G | TE |
| nrpe-125 | chr3 | 10810854 | G->T | TE |
| nrpe-125 | chr1 | 16201810 | C->A | TE |
| nrpe-125 | chr4 | 13650177 | T->C | IG |
| nrpe-125 | chr3 | 15581354 | T->C | TE |
| nrpe-125 | chr2 | 8032281 | G->C | Intron |
| nrpe-125 | chr2 | 3971994 | C->A | TE |
| nrpe-125 | chr2 | 17352304 | T->C | Intron |
| nrpe-125 | chr4 | 2180386 | G->C | TE |
| nrpe-125 | chr1 | 13253396 | A->C | IG |
| nrpe-125 | chr5 | 26073293 | C->G | IG |
| nrpe-125 | chr5 | 2374856 | A->T | TE |
| nrpe-125 | chr1 | 15019401 | T->A | IG |
| nrpe-125 | chr1 | 22887119 | A->G | ncRNA |
| nrpe-125 | chr1 | 23161017 | G->T | Intron |
| nrpe-125 | chr5 | 11817562 | G->A | TE |
| nrpe-125 | chr4 | 1311108 | G->A | IG |
| nrpe-125 | chr1 | 16527543 | A->G | TE |
| nrpe-125 | chr4 | 4289796 | C->A | TE |
| nrpe-125 | chr1 | 4757297 | T->G | Coding |
| nrpe-125 | chr5 | 26751852 | G->T | IG |
| nrpe-125 | chr4 | 7375584 | C->T | TE |
| nrpe-125 | chr5 | 3724160 | T->A | IG |
| nrpe-125 | chr1 | 15651432 | T->A | Intron |
| nrpe-125 | chr2 | 10792370 | C->A | IG |
| nrpe-125 | chr3 | 9411253 | A->G | IG |
| nrpe-125 | chr5 | 14601948 | C->A | IG |
| nrpe-125 | chr2 | 1634955 | G->A | TE |
| nrpe-125 | chr5 | 15225382 | G->A | ncRNA |
| nrpe-125 | chr4 | 13278276 | G->C | IG |
| nrpe-125 | chr1 | 25796421 | T->G | Coding |
| nrpe-125 | chr4 | 14782665 | C->G | IG |
| nrpe-125 | chr1 | 15793100 | G->T | TE |
| nrpe-125 | chr3 | 14976237 | A->G | TE |
| nrpe-125 | chr4 | 4031526 | C->T | TE |
| nrpe-125 | chr3 | 14824729 | C->T | TE |
| nrpe-125 | chr1 | 13173216 | C->A | TE |
| nrpe-125 | chr4 | 1285300 | T->C | Coding |
| nrpe-125 | chr4 | 17997602 | G->A | IG |
| nrpe-125 | chr1 | 9706374 | G->T | IG |
| nrpe-125 | chr2 | 7648418 | T->C | Coding |
| nrpe-125 | chr4 | 1365761 | G->C | IG |
| nrpe-125 | chr2 | 5199506 | T->C | TE |
| nrpe-125 | chr1 | 14687511 | T->G | IG |
| nrpe-125 | chr3 | 4343840 | A->T | IG |
| nrpe-125 | chr2 | 3029039 | G->T | Coding |
| nrpe-125 | chr2 | 9458792 | A->G | Coding |
| nrpe-125 | chr1 | 12269380 | A->T | Coding |
| nrpe-125 | chr3 | 12313710 | G->A | TE |
| nrpe-125 | chr5 | 21970145 | T->A | IG |
| nrpe-125 | chr3 | 13164811 | A->G | TE |
| nrpe-125 | chr2 | 3923520 | A->T | TE |
| nrpe-125 | chr2 | 2569809 | C->A | TE |
| nrpe-125 | chr1 | 18613916 | C->A | Coding |
| nrpe-125 | chr5 | 24042644 | C->G | Coding |
| nrpe-125 | chr4 | 8257296 | C->A | Intron |
| nrpe-125 | chr5 | 11267401 | C->A | IG |
| nrpe-125 | chr5 | 15505196 | A->T | TE |
| nrpe-125 | chr5 | 22066056 | A->G | Coding |
| nrpe-125 | chr3 | 13709502 | T->C | TE |
| nrpe-125 | chr5 | 20427081 | C->T | TE |
| nrpe-125 | chr4 | 1285301 | A->C | Coding |
| nrpe-125 | chr5 | 15829757 | G->A | IG |
| nrpe-125 | chr1 | 16963101 | T->C | TE |
| nrpe-125 | chr4 | 1353354 | A->G | pseudogene |
| nrpe-125 | chr1 | 822095 | T->C | TE |
| nrpe-125 | chr1 | 3069072 | T->A | TE |
| nrpe-125 | chr3 | 7040503 | G->T | IG |
| nrpe-125 | chr4 | 1099437 | C->T | TE |
| nrpe-125 | chr5 | 23137303 | G->A | TE |
| nrpe-125 | chr5 | 19847370 | A->G | UTR |
| nrpe-125 | chr1 | 13185902 | C->T | TE |
| nrpe-125 | chr2 | 2569715 | G->A | TE |
| nrpe-125 | chr3 | 12313587 | C->T | TE |
| nrpe-125 | chr2 | 11256501 | A->G | Intron |
| nrpe-125 | chr2 | 2829472 | C->A | TE |
| nrpe-125 | chr2 | 4815064 | A->C | TE |
| nrpe-125 | chr3 | 18005767 | T->G | IG |
| nrpe-125 | chr3 | 16194158 | G->A | Coding |
| nrpe-125 | chr3 | 5086564 | C->A | Coding |
| nrpe-125 | chr2 | 18749577 | C->A | IG |
| nrpe-125 | chr4 | 1395895 | A->C | TE |
| nrpe-125 | chr2 | 17272630 | T->A | Intron |
| nrpe-125 | chr1 | 4266051 | T->G | IG |
| nrpe-125 | chr3 | 14414282 | G->T | TE |
| nrpe-125 | chr4 | 1340908 | T->G | ncRNA |
| nrpe-125 | chr1 | 29191794 | T->A | IG |
| nrpe-125 | chr3 | 12437188 | G->T | TE |
| nrpe-125 | chr2 | 4331112 | A->G | IG |
| nrpe-125 | chr5 | 14013081 | A->T | TE |
| nrpe-125 | chr5 | 8748936 | C->T | TE |
| nrpe-125 | chr1 | 13350476 | C->T | IG |
| nrpe-125 | chr1 | 6059980 | A->G | Intron |
| nrpe-125 | chr1 | 15493224 | A->T | TE |
| nrpe-125 | chr4 | 4372890 | G->C | TE |
| nrpe-125 | chr4 | 2259564 | G->T | IG |
| nrpe-125 | chr2 | 1811568 | T->C | TE |
| nrpe-125 | chr1 | 5967444 | A->C | TE |
| nrpe-125 | chr5 | 10165055 | G->C | TE |
| nrpe-125 | chr2 | 7902278 | G->T | IG |
| nrpe-125 | chr5 | 6178217 | A->G | UTR |
| nrpe-125 | chr3 | 13750452 | G->A | IG |
| nrpe-125 | chr3 | 19699921 | T->C | IG |
| nrpe-125 | chr2 | 2047006 | G->A | TE |
| nrpe-125 | chr4 | 4008338 | G->A | IG |
| nrpe-125 | chr1 | 26899523 | T->C | IG |
| nrpe-125 | chr3 | 19188790 | C->G | IG |
| nrpe-125 | chr5 | 3335074 | G->C | Coding |
| nrpe-125 | chr1 | 16136717 | T->C | Intron |
| nrpe-125 | chr2 | 8037373 | A->T | IG |
| nrpe-125 | chr5 | 17143959 | G->A | Coding |
| nrpe-125 | chr3 | 5198098 | A->T | Intron |
| nrpe-125 | chr4 | 14795657 | C->A | IG |
| nrpe-125 | chr1 | 9999402 | G->T | Coding |
| nrpe-125 | chr5 | 9634637 | C->T | Coding |
| nrpe-125 | chr1 | 10829236 | C->G | Coding |
| nrpe-125 | chr3 | 21494300 | A->T | Coding |
| nrpe-125 | chr3 | 485272 | G->C | Coding |
| nrpe-125 | chr1 | 23108246 | C->A | Intron |
| nrpe-125 | chr4 | 6936616 | C->G | TE |
| nrpe-125 | chr3 | 4561148 | A->G | UTR |
| nrpe-125 | chr5 | 19409342 | A->C | UTR |
| nrpe-125 | chr4 | 8905021 | C->G | TE |
| nrpe-125 | chr4 | 1408856 | C->G | Coding |
| nrpe-125 | chr2 | 15230027 | A->G | UTR |
| nrpe-125 | chr1 | 1856619 | A->G | Intron |
| ddc-125 | chr3 | 12846647 | G->A | TE |
| ddc-125 | chr1 | 28578222 | T->C | Coding |
| ddc-125 | chr5 | 4309453 | T->A | Intron |
| ddc-125 | chr5 | 7094544 | T->C | IG |
| ddc-125 | chr1 | 24024976 | G->A | Intron |
| ddc-125 | chr2 | 13125545 | A->C | Intron |
| ddc-125 | chr1 | 2084246 | G->C | Intron |
| ddc-125 | chr5 | 18773812 | C->A | IG |
| ddc-125 | chr5 | 10084958 | A->T | IG |
| ddc-125 | chr1 | 30109847 | T->G | Coding |
| ddc-125 | chr1 | 21349107 | G->A | TE |
| ddc-125 | chr2 | 16774728 | A->G | IG |
| ddc-125 | chr3 | 3167328 | A->T | Coding |
| ddc-125 | chr2 | 9378669 | G->C | IG |
| ddc-125 | chr2 | 16825005 | C->A | IG |
| ddc-125 | chr3 | 7409442 | C->T | UTR |
| ddc-125 | chr2 | 8030810 | A->G | IG |
| ddc-125 | chr5 | 12125597 | A->G | TE |
| ddc-125 | chr1 | 21953480 | G->T | IG |
| ddc-125 | chr2 | 6135475 | G->A | TE |
| ddc-125 | chr1 | 11464939 | A->G | IG |
| ddc-125 | chr3 | 2918490 | C->A | IG |
| ddc-125 | chr1 | 24023720 | G->A | UTR |
| ddc-125 | chr3 | 12524321 | G->A | TE |
| ddc-125 | chr1 | 9338191 | G->T | IG |
| ddc-125 | chr5 | 7685200 | T->C | IG |
| ddc-125 | chr5 | 13266290 | G->A | TE |
| ddc-125 | chr3 | 20544119 | C->T | Coding |
| ddc-125 | chr1 | 25260780 | G->T | IG |
| ddc-125 | chr5 | 13268405 | G->T | TE |
| ddc-125 | chr3 | 13840750 | A->G | TE |
| ddc-125 | chr1 | 29002257 | C->G | ncRNA |
| ddc-125 | chr4 | 9189133 | G->T | TE |
| ddc-125 | chr2 | 17805505 | A->G | Coding |
| ddc-125 | chr2 | 7640323 | G->T | IG |
| ddc-125 | chr3 | 10416993 | G->T | IG |
| ddc-125 | chr3 | 12869548 | T->A | TE |
| ddc-125 | chr5 | 4733770 | G->C | UTR |
| ddc-125 | chr2 | 3558260 | C->A | TE |
| ddc-125 | chr5 | 26041311 | A->G | Coding |
| ddc-125 | chr3 | 13945274 | A->T | TE |
| ddc-125 | chr2 | 9488543 | A->G | IG |
| ddc-125 | chr1 | 13872333 | G->T | IG |
| ddc-125 | chr3 | 22733338 | A->G | UTR |
| ddc-125 | chr2 | 14474355 | T->C | Coding |
| ddc-125 | chr1 | 15421474 | A->T | TE |
| ddc-125 | chr2 | 6374206 | C->T | TE |
| ddc-125 | chr2 | 17345248 | T->A | Intron |
| ddc-125 | chr3 | 12247101 | A->G | TE |
| ddc-125 | chr5 | 11483042 | C->T | TE |
| ddc-125 | chr5 | 743138 | G->C | IG |
| ddc-125 | chr3 | 13806841 | G->A | TE |
| ddc-125 | chr5 | 14826153 | C->A | IG |
| ddc-125 | chr3 | 9104814 | A->G | Intron |
| ddc-125 | chr1 | 23008667 | G->C | IG |
| ddc-125 | chr3 | 14260310 | C->A | TE |
| ddc-125 | chr1 | 15554121 | G->A | TE |
| ddc-125 | chr4 | 2975510 | A->C | TE |
| ddc-125 | chr3 | 651702 | T->C | Intron |
| ddc-125 | chr5 | 17405697 | A->G | TE |
| ddc-125 | chr4 | 4914913 | G->C | TE |
| ddc-125 | chr4 | 1638693 | G->A | IG |
| ddc-125 | chr5 | 19078658 | A->G | Intron |
| ddc-125 | chr3 | 12215946 | G->T | TE |
| ddc-125 | chr5 | 5814684 | T->G | IG |
| ddc-125 | chr3 | 8466324 | C->T | IG |
| ddc-125 | chr4 | 13391589 | C->A | Coding |
| ddc-125 | chr2 | 11397684 | T->G | TE |
| ddc-125 | chr5 | 7272018 | C->A | IG |
| ddc-125 | chr2 | 4229372 | G->A | IG |
| ddc-125 | chr4 | 17132941 | A->C | Intron |
| ddc-125 | chr4 | 7781851 | G->T | Coding |
| ddc-125 | chr3 | 12716810 | G->A | TE |
| ddc-125 | chr1 | 16631604 | G->C | TE |
| ddc-125 | chr1 | 29284642 | C->T | IG |
| ddc-125 | chr4 | 3992388 | C->A | TE |
| ddc-125 | chr3 | 13787314 | T->G | IG |
| ddc-125 | chr1 | 22632453 | G->A | IG |
| ddc-125 | chr1 | 26060860 | A->T | IG |
| ddc-125 | chr1 | 13506960 | G->T | TE |
| ddc-125 | chr4 | 2744311 | C->G | Coding |
| ddc-125 | chr1 | 16628678 | C->T | TE |
| ddc-125 | chr5 | 5152894 | C->G | Intron |
| ddc-125 | chr4 | 13719900 | C->T | Coding |
| ddc-125 | chr3 | 4249665 | G->T | Coding |
| ddc-125 | chr1 | 8567314 | T->G | Intron |
| ddc-125 | chr3 | 14916406 | A->G | TE |
| ddc-125 | chr1 | 14094300 | A->G | TE |
| ddc-125 | chr1 | 10407528 | G->T | Coding |
| ddc-125 | chr1 | 13100058 | G->T | TE |
| ddc-125 | chr2 | 5180169 | A->G | IG |
| ddc-125 | chr5 | 24813945 | C->T | Coding |
| ddc-125 | chr1 | 20534312 | C->G | pseudogene |
| ddc-125 | chr5 | 5549166 | C->G | Intron |
| ddc-125 | chr3 | 7039912 | G->A | Coding |
| ddc-125 | chr2 | 6659366 | G->A | TE |
| ddc-125 | chr1 | 21455443 | C->T | TE |
| ddc-125 | chr5 | 15044539 | C->A | IG |
| ddc-125 | chr1 | 2394372 | A->C | IG |
| ddc-125 | chr3 | 15932408 | T->C | Coding |
| ddc-125 | chr1 | 12840680 | A->G | TE |
| ddc-125 | chr3 | 16936238 | T->A | Coding |
| ddc-125 | chr3 | 7668337 | C->T | Coding |
| ddc-125 | chr1 | 2649477 | T->C | IG |
| ddc-125 | chr4 | 4016499 | A->G | TE |
| ddc-125 | chr5 | 10885861 | G->T | TE |
| ddc-125 | chr3 | 22837850 | C->G | Coding |
| ddc-125 | chr3 | 12074435 | A->G | IG |
| ddc-125 | chr1 | 16171909 | G->A | TE |
| ddc-125 | chr2 | 16850930 | C->A | IG |
| ddc-125 | chr4 | 15909750 | T->A | Intron |
| ddc-125 | chr5 | 12344182 | T->A | pseudogene |
| ddc-125 | chr2 | 2905045 | A->T | TE |
| ddc-125 | chr3 | 16668788 | C->A | Intron |
| ddc-125 | chr5 | 26409548 | T->C | Coding |
| ddc-125 | chr1 | 15181718 | G->A | TE |
| ddc-125 | chr5 | 4440615 | G->A | Intron |
| ddc-125 | chr5 | 77534 | A->G | Coding |
| ddc-125 | chr3 | 15150045 | T->C | TE |
| ddc-125 | chr1 | 7339476 | A->G | IG |
| ddc-125 | chr3 | 21558762 | G->C | IG |
| ddc-125 | chr1 | 1108002 | T->C | Coding |
| ddc-125 | chr2 | 2031495 | G->T | TE |
| ddc-125 | chr2 | 1790292 | A->G | TE |
| ddc-125 | chr5 | 23854399 | C->G | Coding |
| ddc-125 | chr3 | 21551342 | T->C | IG |
| ddc-125 | chr3 | 12921886 | A->G | TE |
| ddc-125 | chr1 | 14461419 | G->T | TE |
| ddc-125 | chr4 | 3002960 | C->G | TE |
| ddc-125 | chr5 | 25779888 | T->C | Coding |
| ddc-125 | chr4 | 17774 | A->G | TE |
| ddc-125 | chr2 | 303579 | C->A | TE |
| ddc-125 | chr3 | 1073621 | C->T | Intron |
| ddc-125 | chr3 | 10983205 | G->C | Coding |
| ddc-125 | chr1 | 29097880 | G->T | Coding |
| ddc-125 | chr2 | 1173937 | A->G | Coding |
| ddc-125 | chr2 | 3613671 | G->C | IG |
| ddc-125 | chr4 | 11518408 | C->T | Coding |
| ddc-125 | chr4 | 10574586 | C->A | IG |
| ddc-125 | chr3 | 13229391 | C->T | TE |
| ddc-125 | chr5 | 9910201 | T->C | IG |
| ddc-125 | chr1 | 19778712 | C->T | Coding |
| ddc-125 | chr1 | 29497330 | A->G | IG |
| ddc-125 | chr1 | 20853749 | T->C | Intron |
| ddc-125 | chr4 | 14828179 | G->T | IG |
| ddc-125 | chr1 | 12143327 | A->T | TE |
| ddc-125 | chr3 | 11574669 | C->T | TE |
| ddc-125 | chr4 | 14679599 | A->C | Intron |
| ddc-125 | chr4 | 3588896 | A->T | TE |
| ddc-125 | chr5 | 15586535 | G->C | IG |
| ddc-125 | chr1 | 29366079 | A->G | IG |
| ddc-125 | chr5 | 13380105 | A->G | TE |
| ddc-125 | chr3 | 14683901 | G->A | TE |
| ddc-125 | chr4 | 4299762 | T->A | TE |
| ddc-125 | chr3 | 12153243 | A->T | TE |
| ddc-125 | chr5 | 14851535 | C->A | IG |
| ddc-125 | chr2 | 3150640 | A->G | TE |
| ddc-125 | chr5 | 19410328 | T->C | Coding |
| ddc-125 | chr5 | 5369244 | A->T | Coding |
| ddc-125 | chr2 | 17090800 | A->C | TE |
| ddc-125 | chr1 | 19008802 | T->A | Intron |
| ddc-125 | chr3 | 16317556 | G->T | Coding |
| ddc-125 | chr4 | 8833234 | A->G | IG |
| ddc-125 | chr1 | 22111234 | A->G | IG |
| ddc-125 | chr4 | 11931637 | G->A | UTR |
| ddc-125 | chr2 | 46187 | G->A | TE |
| ddc-125 | chr5 | 7244779 | T->A | Intron |
| ddc-125 | chr5 | 592036 | C->T | Coding |
| ddc-125 | chr5 | 12749283 | A->G | TE |
| ddc-125 | chr5 | 22664767 | T->A | UTR |
| ddc-125 | chr2 | 16942890 | A->G | Intron |
| ddc-125 | chr4 | 3700916 | T->A | TE |
| ddc-125 | chr1 | 2452718 | A->C | Coding |
| ddc-125 | chr5 | 15045490 | G->C | Coding |
| ddc-125 | chr4 | 4520302 | G->A | TE |
| ddc-125 | chr4 | 3989501 | G->A | TE |
| ddc-125 | chr2 | 36387 | G->A | TE |
| ddc-125 | chr1 | 16200774 | C->A | TE |
| ddc-125 | chr2 | 1174389 | T->A | TE |
| ddc-125 | chr2 | 2569809 | C->A | TE |
| ddc-125 | chr3 | 22425465 | C->G | Coding |
| ddc-125 | chr2 | 3580505 | A->T | UTR |
| ddc-125 | chr1 | 15188951 | C->T | TE |
| ddc-125 | chr2 | 983668 | T->C | Coding |
| ddc-125 | chr1 | 18132852 | G->T | Coding |
| ddc-125 | chr3 | 22111183 | T->A | Coding |
| ddc-125 | chr5 | 3098380 | A->G | Coding |
| ddc-125 | chr3 | 15550044 | G->A | TE |
| ddc-125 | chr5 | 4787238 | C->G | Intron |
| ddc-125 | chr4 | 3991305 | G->A | TE |
| ddc-125 | chr2 | 9714025 | C->A | IG |
| ddc-125 | chr1 | 22045703 | A->G | Coding |
| ddc-125 | chr5 | 13367901 | G->C | TE |
| ddc-125 | chr1 | 18854230 | A->G | TE |
| ddc-125 | chr2 | 2797886 | G->A | TE |
| ddc-125 | chr5 | 12346820 | G->A | pseudogene |
| ddc-125 | chr4 | 13545254 | A->G | Coding |
| ddc-125 | chr4 | 16525284 | C->A | IG |
| ddc-125 | chr5 | 740518 | T->A | IG |
| ddc-125 | chr4 | 3739496 | G->A | TE |
| ddc-125 | chr3 | 11172241 | C->T | TE |
| ddc-125 | chr3 | 14054504 | G->C | TE |
| ddc-125 | chr2 | 17991555 | T->C | Coding |
| ddc-125 | chr4 | 847426 | C->G | IG |
| ddc-125 | chr3 | 21333041 | T->C | Coding |
| ddc-125 | chr3 | 416512 | G->A | Coding |
| ddc-125 | chr3 | 13133776 | A->T | TE |
| ddc-125 | chr5 | 11719134 | A->G | TE |
| ddc-125 | chr4 | 11474689 | A->G | IG |
| ddc-125 | chr3 | 16626002 | A->G | IG |
| ddc-125 | chr4 | 15085587 | C->T | Intron |
| ddc-125 | chr2 | 11486001 | G->A | Coding |
| ddc-125 | chr1 | 26513289 | C->A | Coding |
| ddc-125 | chr3 | 13810597 | A->C | IG |
| ddc-125 | chr2 | 3629020 | T->G | IG |
| ddc-125 | chr5 | 7037485 | T->A | TE |
| ddc-125 | chr4 | 1794708 | C->T | TE |
| ddc-125 | chr3 | 20465697 | G->C | Coding |
| ddc-125 | chr1 | 9267492 | A->G | IG |
| ddc-125 | chr1 | 10090435 | G->T | ncRNA |
| ddc-125 | chr4 | 10427224 | C->A | TE |
| ddc-125 | chr4 | 17837930 | T->A | Intron |
| ddc-125 | chr3 | 20909376 | G->A | Coding |
| ddc-125 | chr1 | 193751 | A->G | TE |
| ddc-125 | chr4 | 3500290 | A->G | TE |
| ddc-125 | chr3 | 12900506 | C->T | TE |
| ddc-125 | chr1 | 14462923 | G->T | TE |
| ddc-125 | chr1 | 16521566 | G->A | TE |
| ddc-125 | chr3 | 11876405 | G->A | TE |
| ddc-125 | chr5 | 9509365 | C->A | Coding |
| ddc-125 | chr5 | 5262427 | C->A | Intron |
| ddc-125 | chr4 | 15382058 | G->T | IG |
| ddc-125 | chr3 | 20383942 | T->C | IG |
| ddc-125 | chr5 | 969901 | A->C | IG |
| ddc-125 | chr5 | 15041815 | T->C | TE |
| ddc-125 | chr4 | 5988670 | G->A | TE |
| ddc-125 | chr1 | 4071353 | T->C | Coding |
| ddc-125 | chr5 | 9163689 | T->C | Coding |
| ddc-125 | chr1 | 27852175 | G->T | Coding |
| ddc-125 | chr3 | 7219158 | C->G | IG |
| ddc-125 | chr5 | 10790376 | A->G | TE |
| ddc-125 | chr3 | 4494864 | A->G | TE |
| ddc-125 | chr1 | 9090698 | T->A | Coding |
| ddc-125 | chr2 | 5076964 | G->T | TE |
| ddc-125 | chr5 | 11778313 | C->T | TE |
| ddc-125 | chr1 | 5047318 | C->A | IG |
| ddc-125 | chr2 | 8941166 | G->T | UTR |
| ddc-125 | chr5 | 11705470 | G->C | IG |
| ddc-125 | chr5 | 11053667 | A->G | TE |
| ddc-125 | chr2 | 1376 | C->T | Intron |
| ddc-125 | chr4 | 3581322 | C->G | TE |
| ddc-125 | chr3 | 11683276 | G->T | TE |
| ddc-125 | chr5 | 9255065 | C->G | UTR |
| ddc-125 | chr4 | 3851899 | C->A | TE |
| ddc-125 | chr2 | 9527212 | G->A | Coding |
| ddc-150 | chr3 | 11876405 | G->A | TE |
| ddc-150 | chr2 | 1769570 | T->A | IG |
| ddc-150 | chr1 | 10090435 | G->T | ncRNA |
| ddc-150 | chr5 | 7685200 | T->C | IG |
| ddc-150 | chr5 | 7094544 | T->C | IG |
| ddc-150 | chr1 | 20534312 | C->G | pseudogene |
| ddc-150 | chr4 | 17774 | A->G | TE |
| ddc-150 | chr5 | 14809128 | A->G | TE |
| ddc-150 | chr4 | 16739337 | C->A | Coding |
| ddc-150 | chr1 | 14461419 | G->T | TE |
| ddc-150 | chr5 | 10084958 | A->T | IG |
| ddc-150 | chr2 | 5039987 | C->T | TE |
| ddc-150 | chr5 | 10790376 | A->G | TE |
| ddc-150 | chr4 | 3873081 | C->T | TE |
| ddc-150 | chr2 | 17090800 | A->C | TE |
| ddc-150 | chr2 | 4214755 | C->A | TE |
| ddc-150 | chr4 | 14679599 | A->C | Intron |
| ddc-150 | chr5 | 12125597 | A->G | TE |
| ddc-150 | chr2 | 1174389 | T->A | TE |
| ddc-150 | chr1 | 20853749 | T->C | Intron |
| ddc-150 | chr1 | 8567314 | T->G | Intron |
| ddc-150 | chr4 | 10427224 | C->A | TE |
| ddc-150 | chr1 | 24023720 | G->A | UTR |
| ddc-150 | chr5 | 13372832 | C->T | TE |
| ddc-150 | chr3 | 12869548 | T->A | TE |
| ddc-150 | chr4 | 8157138 | A->G | IG |
| ddc-150 | chr1 | 24824002 | C->A | IG |
| ddc-150 | chr5 | 15586535 | G->C | IG |
| ddc-150 | chr4 | 9189133 | G->T | TE |
| ddc-150 | chr4 | 3581322 | C->G | TE |
| ddc-150 | chr5 | 14819269 | C->G | UTR |
| ddc-150 | chr3 | 12716810 | G->A | TE |
| ddc-150 | chr5 | 9509365 | C->A | Coding |
| ddc-150 | chr1 | 18854230 | A->G | TE |
| ddc-150 | chr1 | 13518543 | A->C | TE |
| ddc-150 | chr1 | 9267492 | A->G | IG |
| ddc-150 | chr1 | 16521566 | G->A | TE |
| ddc-150 | chr3 | 14054504 | G->C | TE |
| ddc-150 | chr2 | 4229372 | G->A | IG |
| ddc-150 | chr2 | 9378669 | G->C | IG |
| ddc-150 | chr2 | 3150640 | A->G | TE |
| ddc-150 | chr5 | 14809014 | G->T | TE |
| ddc-150 | chr2 | 7640323 | G->T | IG |
| ddc-150 | chr4 | 3841310 | G->C | TE |
| ddc-150 | chr3 | 22111183 | T->A | Coding |
| ddc-150 | chr3 | 14195978 | A->C | Coding |
| ddc-150 | chr5 | 969901 | A->C | IG |
| ddc-150 | chr3 | 3167328 | A->T | Coding |
| ddc-150 | chr4 | 11760413 | A->G | Intron |
| ddc-150 | chr3 | 10416993 | G->T | IG |
| ddc-150 | chr1 | 15180116 | A->T | TE |
| ddc-150 | chr5 | 740518 | T->A | IG |
| ddc-150 | chr5 | 5549166 | C->G | Intron |
| ddc-150 | chr4 | 1638693 | G->A | IG |
| ddc-150 | chr3 | 14683901 | G->A | TE |
| ddc-150 | chr1 | 28578222 | T->C | Coding |
| ddc-150 | chr5 | 9910201 | T->C | IG |
| ddc-150 | chr3 | 20544119 | C->T | Coding |
| ddc-150 | chr3 | 7219158 | C->G | IG |
| ddc-150 | chr3 | 15150045 | T->C | TE |
| ddc-150 | chr5 | 7244779 | T->A | Intron |
| ddc-150 | chr4 | 4299762 | T->A | TE |
| ddc-150 | chr5 | 22664767 | T->A | UTR |
| ddc-150 | chr1 | 11464939 | A->G | IG |
| ddc-150 | chr3 | 22971317 | G->A | UTR |
| ddc-150 | chr3 | 11574669 | C->T | TE |
| ddc-150 | chr2 | 8941166 | G->T | UTR |
| ddc-150 | chr3 | 8525642 | A->G | IG |
| ddc-150 | chr1 | 29366079 | A->G | IG |
| ddc-150 | chr3 | 20465697 | G->C | Coding |
| ddc-150 | chr3 | 416512 | G->A | Coding |
| ddc-150 | chr1 | 15208986 | C->G | TE |
| ddc-150 | chr2 | 2275716 | G->A | TE |
| ddc-150 | chr3 | 8466324 | C->T | IG |
| ddc-150 | chr1 | 13517770 | T->G | TE |
| ddc-150 | chr4 | 8833234 | A->G | IG |
| ddc-150 | chr3 | 16668788 | C->A | Intron |
| ddc-150 | chr2 | 3558260 | C->A | TE |
| ddc-150 | chr3 | 14196557 | G->A | UTR |
| ddc-150 | chr1 | 7339476 | A->G | IG |
| ddc-150 | chr1 | 2342824 | G->A | Coding |
| ddc-150 | chr1 | 29097880 | G->T | Coding |
| ddc-150 | chr4 | 3985208 | G->T | IG |
| ddc-150 | chr1 | 22045703 | A->G | Coding |
| ddc-150 | chr2 | 3549013 | C->T | TE |
| ddc-150 | chr1 | 27469354 | G->T | IG |
| ddc-150 | chr5 | 12749283 | A->G | TE |
| ddc-150 | chr2 | 2031495 | G->T | TE |
| ddc-150 | chr5 | 1042718 | C->T | IG |
| ddc-150 | chr2 | 5039988 | G->T | TE |
| ddc-150 | chr1 | 193751 | A->G | TE |
| ddc-150 | chr1 | 26513289 | C->A | Coding |
| ddc-150 | chr2 | 5076964 | G->T | TE |
| ddc-150 | chr5 | 9255065 | C->G | UTR |
| ddc-150 | chr4 | 13582861 | C->A | Coding |
| ddc-150 | chr1 | 19327483 | A->T | TE |
| ddc-150 | chr4 | 13545254 | A->G | Coding |
| ddc-150 | chr5 | 8020114 | T->A | UTR |
| ddc-150 | chr2 | 8030810 | A->G | IG |
| ddc-150 | chr1 | 13010540 | G->A | TE |
| ddc-150 | chr1 | 6738709 | C->T | Coding |
| ddc-150 | chr2 | 1033 | C->T | Coding |
| ddc-150 | chr2 | 36387 | G->A | TE |
| ddc-150 | chr2 | 2363801 | C->A | TE |
| ddc-150 | chr4 | 15085587 | C->T | Intron |
| ddc-150 | chr1 | 12143327 | A->T | TE |
| ddc-150 | chr3 | 12252331 | C->G | TE |
| ddc-150 | chr3 | 16317556 | G->T | Coding |
| ddc-150 | chr1 | 19008802 | T->A | Intron |
| ddc-150 | chr5 | 19410328 | T->C | Coding |
| ddc-150 | chr5 | 9152418 | G->T | Coding |
| ddc-150 | chr3 | 16936238 | T->A | Coding |
| ddc-150 | chr2 | 13208426 | G->C | IG |
| ddc-150 | chr2 | 10335146 | A->G | Coding |
| ddc-150 | chr1 | 15421474 | A->T | TE |
| ddc-150 | chr1 | 14094300 | A->G | TE |
| ddc-150 | chr3 | 22733338 | A->G | UTR |
| ddc-150 | chr5 | 4309453 | T->A | Intron |
| ddc-150 | chr1 | 13506960 | G->T | TE |
| ddc-150 | chr3 | 1073621 | C->T | Intron |
| ddc-150 | chr5 | 25779888 | T->C | Coding |
| ddc-150 | chr4 | 1794708 | C->T | TE |
| ddc-150 | chr2 | 16942890 | A->G | Intron |
| ddc-150 | chr5 | 9163689 | T->C | Coding |
| ddc-150 | chr3 | 6699947 | A->T | Coding |
| ddc-150 | chr3 | 10983205 | G->C | Coding |
| ddc-150 | chr1 | 30109847 | T->G | Coding |
| ddc-150 | chr2 | 16825005 | C->A | IG |
| ddc-150 | chr5 | 20741722 | A->T | IG |
| ddc-150 | chr4 | 3598621 | G->T | TE |
| ddc-150 | chr2 | 1790292 | A->G | TE |
| ddc-150 | chr3 | 13945274 | A->T | TE |
| ddc-150 | chr1 | 15098415 | T->C | TE |
| ddc-150 | chr5 | 5369244 | A->T | Coding |
| ddc-150 | chr3 | 21558762 | G->C | IG |
| ddc-150 | chr5 | 743138 | G->C | IG |
| ddc-150 | chr1 | 9338191 | G->T | IG |
| ddc-150 | chr2 | 3951070 | A->G | TE |
| ddc-150 | chr3 | 14195926 | G->A | Coding |
| ddc-150 | chr2 | 16774728 | A->G | IG |
| ddc-150 | chr2 | 1376 | C->T | Intron |
| ddc-150 | chr4 | 3500290 | A->G | TE |
| ddc-150 | chr1 | 5047318 | C->A | IG |
| ddc-150 | chr3 | 1752548 | T->C | TE |
| ddc-150 | chr5 | 14810107 | A->T | IG |
| ddc-150 | chr1 | 19778712 | C->T | Coding |
| ddc-150 | chr2 | 2905045 | A->T | TE |
| ddc-150 | chr3 | 5931998 | A->G | IG |
| ddc-150 | chr1 | 29497330 | A->G | IG |
| ddc-150 | chr3 | 13229391 | C->T | TE |
| ddc-150 | chr5 | 26041311 | A->G | Coding |
| ddc-150 | chr4 | 11518408 | C->T | Coding |
| ddc-150 | chr2 | 1173937 | A->G | Coding |
| ddc-150 | chr4 | 17132941 | A->C | Intron |
| ddc-150 | chr4 | 16525284 | C->A | IG |
| ddc-150 | chr3 | 7668337 | C->T | Coding |
| ddc-150 | chr5 | 17207873 | A->C | Coding |
| ddc-150 | chr3 | 14260310 | C->A | TE |
| ddc-150 | chr1 | 15554121 | G->A | TE |
| ddc-150 | chr3 | 7039912 | G->A | Coding |
| ddc-150 | chr3 | 12900506 | C->T | TE |
| ddc-150 | chr3 | 4494864 | A->G | TE |
| ddc-150 | chr2 | 9488543 | A->G | IG |
| ddc-150 | chr5 | 15040420 | A->C | TE |
| ddc-150 | chr1 | 2452718 | A->C | Coding |
| ddc-150 | chr4 | 10574586 | C->A | IG |
| ddc-150 | chr2 | 2797886 | G->A | TE |
| ddc-150 | chr2 | 11486001 | G->A | Coding |
| ddc-150 | chr5 | 24813945 | C->T | Coding |
| ddc-150 | chr3 | 15550044 | G->A | TE |
| ddc-150 | chr1 | 1108002 | T->C | Coding |
| ddc-150 | chr4 | 4914913 | G->C | TE |
| ddc-150 | chr2 | 3580505 | A->T | UTR |
| ddc-150 | chr5 | 26409548 | T->C | Coding |
| ddc-150 | chr4 | 3700916 | T->A | TE |
| ddc-150 | chr1 | 23008667 | G->C | IG |
| ddc-150 | chr2 | 14939455 | A->G | pseudogene |
| ddc-150 | chr5 | 19078658 | A->G | Intron |
| ddc-150 | chr5 | 13266290 | G->A | TE |
| ddc-150 | chr4 | 4822801 | C->A | TE |
| ddc-150 | chr3 | 14916406 | A->G | TE |
| ddc-150 | chr2 | 17805505 | A->G | Coding |
| ddc-150 | chr5 | 12346820 | G->A | pseudogene |
| ddc-150 | chr3 | 651702 | T->C | Intron |
| ddc-150 | chr3 | 9104814 | A->G | Intron |
| ddc-150 | chr2 | 17345248 | T->A | Intron |
| ddc-150 | chr3 | 12524321 | G->A | TE |
| ddc-150 | chr3 | 16626002 | A->G | IG |
| ddc-150 | chr1 | 21210725 | A->T | TE |
| ddc-150 | chr1 | 10407528 | G->T | Coding |
| ddc-150 | chr5 | 10885861 | G->T | TE |
| ddc-150 | chr4 | 5988670 | G->A | TE |
| ddc-150 | chr1 | 16526258 | A->C | TE |
| ddc-150 | chr2 | 11397684 | T->G | TE |
| ddc-150 | chr3 | 13810597 | A->C | IG |
| ddc-150 | chr5 | 3098380 | A->G | Coding |
| ddc-150 | chr5 | 11483042 | C->T | TE |
| ddc-150 | chr5 | 14823231 | T->A | TE |
| ddc-150 | chr1 | 21349107 | G->A | TE |
| ddc-150 | chr3 | 20909376 | G->A | Coding |
| ddc-150 | chr3 | 12846647 | G->A | TE |
| ddc-150 | chr4 | 2744311 | C->G | Coding |
| ddc-150 | chr3 | 22837850 | C->G | Coding |
| ddc-150 | chr5 | 4733770 | G->C | UTR |
| ddc-150 | chr4 | 4520302 | G->A | TE |
| ddc-150 | chr2 | 7454839 | A->G | TE |
| ddc-150 | chr1 | 24024976 | G->A | Intron |
| ddc-150 | chr5 | 15673730 | G->A | TE |
| ddc-150 | chr3 | 13806841 | G->A | TE |
| ddc-150 | chr5 | 23939455 | T->A | IG |
| ddc-150 | chr4 | 3588896 | A->T | TE |
| ddc-150 | chr3 | 11172241 | C->T | TE |
| ddc-150 | chr5 | 15045490 | G->C | Coding |
| ddc-150 | chr3 | 21551342 | T->C | IG |
| ddc-150 | chr3 | 15932408 | T->C | Coding |
| ddc-150 | chr4 | 17837930 | T->A | Intron |
| ddc-150 | chr5 | 7272018 | C->A | IG |
| ddc-150 | chr1 | 2649477 | T->C | IG |
| ddc-150 | chr1 | 12943719 | C->T | TE |
| ddc-150 | chr1 | 16522014 | T->C | TE |
| ddc-150 | chr3 | 4249665 | G->T | Coding |
| ddc-150 | chr4 | 15909750 | T->A | Intron |
| ddc-150 | chr2 | 48429 | C->T | TE |
| ddc-150 | chr1 | 2394372 | A->C | IG |
| ddc-150 | chr5 | 13380105 | A->G | TE |
| ddc-150 | chr4 | 14828179 | G->T | IG |
| ddc-150 | chr4 | 3924602 | T->C | TE |
| ddc-150 | chr4 | 2975510 | A->C | TE |
| ddc-150 | chr5 | 12080123 | A->T | Intron |
| ddc-150 | chr5 | 12037301 | T->C | TE |
| ddc-150 | chr1 | 22111234 | A->G | IG |
| ddc-150 | chr3 | 22425465 | C->G | Coding |
| ddc-150 | chr5 | 4787238 | C->G | Intron |
| ddc-150 | chr5 | 11186717 | G->T | IG |
| ddc-150 | chr5 | 15044539 | C->A | IG |
| ddc-150 | chr3 | 7409442 | C->T | UTR |
| ddc-150 | chr3 | 12153243 | A->T | TE |
| ddc-150 | chr1 | 13872333 | G->T | IG |
| ddc-150 | chr1 | 25260780 | G->T | IG |
| ddc-150 | chr1 | 2084246 | G->C | Intron |
| ddc-150 | chr4 | 3002960 | C->G | TE |
| ddc-150 | chr2 | 4236177 | T->A | TE |
| ddc-150 | chr3 | 12215715 | C->T | TE |
| ddc-150 | chr1 | 16628678 | C->T | TE |
| ddc-175 | chr3 | 14205735 | T->A | IG |
| ddc-175 | chr1 | 29366079 | A->G | IG |
| ddc-175 | chr2 | 5076964 | G->T | TE |
| ddc-175 | chr1 | 20853749 | T->C | Intron |
| ddc-175 | chr4 | 15909750 | T->A | Intron |
| ddc-175 | chr3 | 21551342 | T->C | IG |
| ddc-175 | chr1 | 22632453 | G->A | IG |
| ddc-175 | chr3 | 14683901 | G->A | TE |
| ddc-175 | chr5 | 13372832 | C->T | TE |
| ddc-175 | chr5 | 5814684 | T->G | IG |
| ddc-175 | chr4 | 1638693 | G->A | IG |
| ddc-175 | chr2 | 4229372 | G->A | IG |
| ddc-175 | chr5 | 23939455 | T->A | IG |
| ddc-175 | chr2 | 3580505 | A->T | UTR |
| ddc-175 | chr3 | 12869548 | T->A | TE |
| ddc-175 | chr4 | 2975510 | A->C | TE |
| ddc-175 | chr3 | 14213023 | G->A | IG |
| ddc-175 | chr4 | 11931637 | G->A | UTR |
| ddc-175 | chr1 | 14094300 | A->G | TE |
| ddc-175 | chr5 | 26041311 | A->G | Coding |
| ddc-175 | chr4 | 13719900 | C->T | Coding |
| ddc-175 | chr4 | 3588896 | A->T | TE |
| ddc-175 | chr3 | 22837850 | C->G | Coding |
| ddc-175 | chr4 | 4914913 | G->C | TE |
| ddc-175 | chr4 | 4520302 | G->A | TE |
| ddc-175 | chr1 | 15421474 | A->T | TE |
| ddc-175 | chr1 | 21953480 | G->T | IG |
| ddc-175 | chr3 | 22425465 | C->G | Coding |
| ddc-175 | chr5 | 12125597 | A->G | TE |
| ddc-175 | chr3 | 8466324 | C->T | IG |
| ddc-175 | chr4 | 17774 | A->G | TE |
| ddc-175 | chr1 | 2649477 | T->C | IG |
| ddc-175 | chr2 | 1376 | C->T | Intron |
| ddc-175 | chr3 | 12900506 | C->T | TE |
| ddc-175 | chr2 | 1790292 | A->G | TE |
| ddc-175 | chr1 | 30109847 | T->G | Coding |
| ddc-175 | chr3 | 4249665 | G->T | Coding |
| ddc-175 | chr2 | 303579 | C->A | TE |
| ddc-175 | chr1 | 10090435 | G->T | ncRNA |
| ddc-175 | chr5 | 14851535 | C->A | IG |
| ddc-175 | chr3 | 2918490 | C->A | IG |
| ddc-175 | chr2 | 17991555 | T->C | Coding |
| ddc-175 | chr1 | 15136985 | G->T | IG |
| ddc-175 | chr5 | 14826153 | C->A | IG |
| ddc-175 | chr5 | 21199445 | C->A | Coding |
| ddc-175 | chr4 | 11760413 | A->G | Intron |
| ddc-175 | chr3 | 12153243 | A->T | TE |
| ddc-175 | chr5 | 9255065 | C->G | UTR |
| ddc-175 | chr3 | 15715591 | C->T | TE |
| ddc-175 | chr4 | 10574586 | C->A | IG |
| ddc-175 | chr1 | 12143327 | A->T | TE |
| ddc-175 | chr3 | 10983205 | G->C | Coding |
| ddc-175 | chr3 | 13840750 | A->G | TE |
| ddc-175 | chr5 | 6792206 | G->T | Intron |
| ddc-175 | chr4 | 2744311 | C->G | Coding |
| ddc-175 | chr3 | 15932408 | T->C | Coding |
| ddc-175 | chr1 | 193751 | A->G | TE |
| ddc-175 | chr3 | 1073621 | C->T | Intron |
| ddc-175 | chr4 | 1845176 | G->A | TE |
| ddc-175 | chr3 | 1976239 | G->T | Coding |
| ddc-175 | chr5 | 19078658 | A->G | Intron |
| ddc-175 | chr2 | 3360076 | C->A | Intron |
| ddc-175 | chr5 | 20741722 | A->T | IG |
| ddc-175 | chr1 | 6738709 | C->T | Coding |
| ddc-175 | chr5 | 5152894 | C->G | Intron |
| ddc-175 | chr3 | 16317556 | G->T | Coding |
| ddc-175 | chr3 | 22733338 | A->G | UTR |
| ddc-175 | chr1 | 29002257 | C->G | ncRNA |
| ddc-175 | chr3 | 19125839 | G->A | Coding |
| ddc-175 | chr1 | 16631604 | G->C | TE |
| ddc-175 | chr3 | 20465697 | G->C | Coding |
| ddc-175 | chr2 | 1173937 | A->G | Coding |
| ddc-175 | chr5 | 24813945 | C->T | Coding |
| ddc-175 | chr1 | 11464939 | A->G | IG |
| ddc-175 | chr5 | 11328810 | G->A | TE |
| ddc-175 | chr4 | 11518408 | C->T | Coding |
| ddc-175 | chr3 | 14260310 | C->A | TE |
| ddc-175 | chr2 | 14939455 | A->G | pseudogene |
| ddc-175 | chr5 | 18773812 | C->A | IG |
| ddc-175 | chr1 | 29497330 | A->G | IG |
| ddc-175 | chr5 | 9910201 | T->C | IG |
| ddc-175 | chr1 | 5047318 | C->A | IG |
| ddc-175 | chr5 | 19410328 | T->C | Coding |
| ddc-175 | chr5 | 9163689 | T->C | Coding |
| ddc-175 | chr5 | 9152418 | G->T | Coding |
| ddc-175 | chr4 | 10427224 | C->A | TE |
| ddc-175 | chr3 | 14195978 | A->C | Coding |
| ddc-175 | chr1 | 2342824 | G->A | Coding |
| ddc-175 | chr3 | 7219158 | C->G | IG |
| ddc-175 | chr2 | 3150640 | A->G | TE |
| ddc-175 | chr1 | 10407528 | G->T | Coding |
| ddc-175 | chr1 | 13872333 | G->T | IG |
| ddc-175 | chr4 | 5988670 | G->A | TE |
| ddc-175 | chr4 | 3598621 | G->T | TE |
| ddc-175 | chr1 | 25260780 | G->T | IG |
| ddc-175 | chr5 | 16524518 | A->C | IG |
| ddc-175 | chr5 | 12031043 | T->C | TE |
| ddc-175 | chr1 | 16628678 | C->T | TE |
| ddc-175 | chr2 | 16774728 | A->G | IG |
| ddc-175 | chr1 | 15297755 | T->C | TE |
| ddc-175 | chr1 | 22045703 | A->G | Coding |
| ddc-175 | chr5 | 12080123 | A->T | Intron |
| ddc-175 | chr1 | 2084246 | G->C | Intron |
| ddc-175 | chr3 | 14210783 | T->C | IG |
| ddc-175 | chr3 | 12524321 | G->A | TE |
| ddc-175 | chr5 | 13266290 | G->A | TE |
| ddc-175 | chr3 | 14054504 | G->C | TE |
| ddc-175 | chr3 | 7409442 | C->T | UTR |
| ddc-175 | chr3 | 6699947 | A->T | Coding |
| ddc-175 | chr1 | 1108002 | T->C | Coding |
| ddc-175 | chr2 | 46187 | G->A | TE |
| ddc-175 | chr5 | 592036 | C->T | Coding |
| ddc-175 | chr3 | 20544119 | C->T | Coding |
| ddc-175 | chr2 | 2363801 | C->A | TE |
| ddc-175 | chr4 | 7819572 | T->A | TE |
| ddc-175 | chr1 | 15136953 | C->G | TE |
| ddc-175 | chr1 | 2452718 | A->C | Coding |
| ddc-175 | chr5 | 7094544 | T->C | IG |
| ddc-175 | chr4 | 3874729 | C->T | TE |
| ddc-175 | chr1 | 12840680 | A->G | TE |
| ddc-175 | chr1 | 13100058 | G->T | TE |
| ddc-175 | chr5 | 7685200 | T->C | IG |
| ddc-175 | chr1 | 26060860 | A->T | IG |
| ddc-175 | chr2 | 7640323 | G->T | IG |
| ddc-175 | chr4 | 13391589 | C->A | Coding |
| ddc-175 | chr3 | 21333041 | T->C | Coding |
| ddc-175 | chr1 | 4071353 | T->C | Coding |
| ddc-175 | chr5 | 7272018 | C->A | IG |
| ddc-175 | chr3 | 12074435 | A->G | IG |
| ddc-175 | chr1 | 15554121 | G->A | TE |
| ddc-175 | chr3 | 5931998 | A->G | IG |
| ddc-175 | chr4 | 3581322 | C->G | TE |
| ddc-175 | chr2 | 11486001 | G->A | Coding |
| ddc-175 | chr3 | 14195926 | G->A | Coding |
| ddc-175 | chr1 | 24023720 | G->A | UTR |
| ddc-175 | chr4 | 17132941 | A->C | Intron |
| ddc-175 | chr1 | 19778712 | C->T | Coding |
| ddc-175 | chr4 | 3700916 | T->A | TE |
| ddc-175 | chr1 | 8567314 | T->G | Intron |
| ddc-175 | chr3 | 16668788 | C->A | Intron |
| ddc-175 | chr1 | 13517770 | T->G | TE |
| ddc-175 | chr1 | 22111234 | A->G | IG |
| ddc-175 | chr2 | 16942890 | A->G | Intron |
| ddc-175 | chr5 | 9509365 | C->A | Coding |
| ddc-175 | chr1 | 27852175 | G->T | Coding |
| ddc-175 | chr2 | 16850930 | C->A | IG |
| ddc-175 | chr2 | 983668 | T->C | Coding |
| ddc-175 | chr2 | 5180169 | A->G | IG |
| ddc-175 | chr1 | 20534312 | C->G | pseudogene |
| ddc-175 | chr1 | 28578222 | T->C | Coding |
| ddc-175 | chr5 | 15040420 | A->C | TE |
| ddc-175 | chr5 | 10075768 | G->A | IG |
| ddc-175 | chr2 | 8030810 | A->G | IG |
| ddc-175 | chr3 | 13810597 | A->C | IG |
| ddc-175 | chr4 | 1668333 | C->T | TE |
| ddc-175 | chr1 | 14261099 | G->A | TE |
| ddc-175 | chr2 | 11397684 | T->G | TE |
| ddc-175 | chr2 | 17345248 | T->A | Intron |
| ddc-175 | chr4 | 3988666 | C->A | TE |
| ddc-175 | chr1 | 23008667 | G->C | IG |
| ddc-175 | chr2 | 2797886 | G->A | TE |
| ddc-175 | chr5 | 5262427 | C->A | Intron |
| ddc-175 | chr4 | 8833234 | A->G | IG |
| ddc-175 | chr1 | 26513289 | C->A | Coding |
| ddc-175 | chr2 | 17090800 | A->C | TE |
| ddc-175 | chr4 | 3500290 | A->G | TE |
| ddc-175 | chr3 | 13784910 | C->G | TE |
| ddc-175 | chr2 | 4236177 | T->A | TE |
| ddc-175 | chr1 | 9267492 | A->G | IG |
| ddc-175 | chr1 | 16521566 | G->A | TE |
| ddc-175 | chr1 | 9338191 | G->T | IG |
| ddc-175 | chr5 | 15041815 | T->C | TE |
| ddc-175 | chr5 | 5369244 | A->T | Coding |
| ddc-175 | chr2 | 9378669 | G->C | IG |
| ddc-175 | chr3 | 11683276 | G->T | TE |
| ddc-175 | chr3 | 12252331 | C->G | TE |
| ddc-175 | chr3 | 13229391 | C->T | TE |
| ddc-175 | chr3 | 20909376 | G->A | Coding |
| ddc-175 | chr4 | 8157138 | A->G | IG |
| ddc-175 | chr1 | 24024976 | G->A | Intron |
| ddc-175 | chr2 | 3342145 | G->A | TE |
| ddc-175 | chr4 | 4822801 | C->A | TE |
| ddc-175 | chr1 | 27469354 | G->T | IG |
| ddc-175 | chr5 | 743138 | G->C | IG |
| ddc-175 | chr3 | 21558762 | G->C | IG |
| ddc-175 | chr2 | 6374206 | C->T | TE |
| ddc-175 | chr3 | 416512 | G->A | Coding |
| ddc-175 | chr4 | 13582861 | C->A | Coding |
| ddc-175 | chr2 | 9714025 | C->A | IG |
| ddc-175 | chr5 | 12749283 | A->G | TE |
| ddc-175 | chr2 | 8941166 | G->T | UTR |
| ddc-175 | chr2 | 13208426 | G->C | IG |
| ddc-175 | chr5 | 10790376 | A->G | TE |
| ddc-175 | chr3 | 11876405 | G->A | TE |
| ddc-175 | chr3 | 14196557 | G->A | UTR |
| ddc-175 | chr1 | 2394372 | A->C | IG |
| ddc-175 | chr5 | 12036167 | T->A | TE |
| ddc-175 | chr3 | 12716810 | G->A | TE |
| ddc-175 | chr2 | 2570128 | C->A | TE |
| ddc-175 | chr3 | 3167328 | A->T | Coding |
| ddc-175 | chr1 | 16526229 | C->T | TE |
| ddc-175 | chr5 | 7037485 | T->A | TE |
| ddc-175 | chr3 | 651702 | T->C | Intron |
| ddc-175 | chr4 | 1794708 | C->T | TE |
| ddc-175 | chr3 | 7039912 | G->A | Coding |
| ddc-175 | chr2 | 2031495 | G->T | TE |
| ddc-175 | chr5 | 11483042 | C->T | TE |
| ddc-175 | chr1 | 29284642 | C->T | IG |
| ddc-175 | chr1 | 7339476 | A->G | IG |
| ddc-175 | chr4 | 11474689 | A->G | IG |
| ddc-175 | chr3 | 13945274 | A->T | TE |
| ddc-175 | chr4 | 10794658 | T->C | Intron |
| ddc-175 | chr2 | 6135475 | G->A | TE |
| ddc-175 | chr5 | 7244779 | T->A | Intron |
| ddc-175 | chr3 | 2485033 | T->A | Intron |
| ddc-175 | chr2 | 17805505 | A->G | Coding |
| ddc-175 | chr4 | 15085587 | C->T | Intron |
| ddc-175 | chr3 | 16626002 | A->G | IG |
| ddc-175 | chr5 | 15044539 | C->A | IG |
| ddc-175 | chr4 | 16739337 | C->A | Coding |
| ddc-175 | chr5 | 4787238 | C->G | Intron |
| ddc-175 | chr5 | 22913612 | C->T | TE |
| ddc-175 | chr4 | 15382058 | G->T | IG |
| ddc-175 | chr3 | 7668337 | C->T | Coding |
| ddc-175 | chr5 | 25779888 | T->C | Coding |
| ddc-175 | chr1 | 29097880 | G->T | Coding |
| ddc-175 | chr3 | 13806841 | G->A | TE |
| ddc-175 | chr5 | 11781915 | G->A | TE |
| ddc-175 | chr5 | 10084958 | A->T | IG |
| ddc-175 | chr4 | 13545254 | A->G | Coding |
| ddc-175 | chr5 | 4440615 | G->A | Intron |
| ddc-175 | chr5 | 15045490 | G->C | Coding |
| ddc-175 | chr4 | 17837930 | T->A | Intron |
| ddc-175 | chr1 | 18854230 | A->G | TE |
| ddc-175 | chr5 | 15673730 | G->A | TE |
| ddc-175 | chr5 | 3554476 | G->T | Intron |
| ddc-175 | chr2 | 16825005 | C->A | IG |
| ddc-175 | chr5 | 23854399 | C->G | Coding |
| ddc-175 | chr4 | 16525284 | C->A | IG |
| ddc-175 | chr5 | 11719134 | A->G | TE |
| ddc-175 | chr2 | 3558260 | C->A | TE |
| ddc-175 | chr1 | 15111512 | C->T | TE |
| ddc-175 | chr5 | 14809014 | G->T | TE |
| ddc-175 | chr1 | 9090698 | T->A | Coding |
| ddc-175 | chr2 | 9488543 | A->G | IG |
| ddc-175 | chr5 | 13380105 | A->G | TE |
| ddc-175 | chr1 | 24824002 | C->A | IG |
| ddc-175 | chr5 | 22664767 | T->A | UTR |
| ddc-175 | chr1 | 19327483 | A->T | TE |
| ddc-175 | chr4 | 4299762 | T->A | TE |
| ddc-175 | chr4 | 14679599 | A->C | Intron |
| ddc-175 | chr3 | 16936238 | T->A | Coding |

Table S3. A list of total insertion and deletion (Indel) mutations in the first batch of

SAD and SUT cells

| Cell line | Chromosome | Position | indel | Region |
| --- | --- | --- | --- | --- |
| nrpe-0 | chr5 | 23142639 | +A | IG |
| nrpe-0 | chr5 | 4879240 | +T | Intron |
| nrpe-0 | chr1 | 20106586 | +A | IG |
| nrpe-0 | chr5 | 21776497 | +A | TE |
| nrpe-0 | chr3 | 4017760 | +T | UTR |
| nrpe-0 | chr4 | 13477939 | +A | Intron |
| nrpe-0 | chr2 | 10069722 | +A | TE |
| nrpe-0 | chr1 | 11685121 | +TC | ncRNA |
| nrpe-0 | chr2 | 9376298 | +T | IG |
| nrpe-0 | chr5 | 21773974 | +A | UTR |
| nrpe-0 | chr1 | 11788309 | +T | IG |
| nrpe-0 | chr1 | 18544697 | +T | IG |
| nrpe-0 | chr5 | 21024837 | +T | IG |
| nrpe-0 | chr4 | 3060777 | +A | IG |
| nrpe-0 | chr5 | 9887368 | +AT | TE |
| nrpe-0 | chr4 | 13069129 | +A | Intron |
| nrpe-0 | chr5 | 20980081 | +A | IG |
| nrpe-0 | chr3 | 14224100 | +T | IG |
| nrpe-0 | chr2 | 3628342 | +T | IG |
| nrpe-0 | chr4 | 3872562 | +AG | TE |
| nrpe-0 | chr3 | 22188674 | +T | UTR |
| nrpe-0 | chr4 | 1338516 | +T | IG |
| ddc-0 | chr4 | 8034762 | +T | IG |
| ddc-0 | chr4 | 14896669 | +T | UTR |
| ddc-0 | chr4 | 4355435 | +GT | IG |
| ddc-0 | chr4 | 4428184 | +G | TE |
| ddc-0 | chr4 | 5892043 | +A | IG |
| ddc-0 | chr5 | 4003800 | +GAGA | Intron |
| ddc-0 | chr2 | 5592598 | +A | TE |
| ddc-0 | chr2 | 3628342 | +T | IG |
| ddc-0 | chr1 | 14592658 | +CCACCACAACAGAACAAA | TE |
| Col-0 | chr4 | 6722397 | +T | TE |
| Col-0 | chr4 | 9140965 | +A | Intron |
| Col-0 | chr2 | 16446401 | +A | IG |
| Col-0 | chr3 | 16999832 | +C | Intron |
| Col-0 | chr2 | 588074 | +T | IG |
| Col-0 | chr5 | 11891268 | +G | IG |
| Col-0 | chr1 | 18918974 | +T | IG |
| Col-0 | chr3 | 17640371 | +AG | IG |
| Col-0 | chr5 | 18884351 | +A | UTR |
| Col-0 | chr2 | 16580523 | +A | Intron |
| Col-0 | chr4 | 11701157 | +A | Intron |
| Col-0 | chr2 | 16928221 | +T | IG |
| Col-0 | chr1 | 26205087 | +T | Intron |
| Col-0 | chr2 | 8414101 | +T | IG |
| Col-0 | chr5 | 17488923 | +A | IG |
| Col-0 | chr3 | 21366019 | +TA | IG |
| Col-0 | chr2 | 19330942 | +TA | IG |
| Col-0 | chr3 | 12940182 | +T | TE |
| Col-0 | chr3 | 16113723 | +A | TE |
| Col-0 | chr1 | 27767199 | +A | IG |
| Col-0 | chr2 | 1307485 | +TA | TE |
| Col-0 | chr4 | 18453753 | +A | IG |
| Col-0 | chr2 | 13871922 | +T | UTR |
| Col-0 | chr2 | 4858706 | +A | IG |
| Col-125 | chr3 | 18158522 | +T | IG |
| Col-125 | chr4 | 89542 | +TA | IG |
| Col-125 | chr1 | 22536586 | +A | Intron |
| Col-125 | chr3 | 21366019 | +TA | IG |
| Col-125 | chr3 | 20148395 | +T | UTR |
| Col-125 | chr1 | 20280235 | +A | Intron |
| Col-125 | chr2 | 13452527 | +TA | Intron |
| Col-125 | chr5 | 20862309 | +T | IG |
| Col-125 | chr3 | 20260686 | +A | IG |
| Col-125 | chr4 | 16524937 | +AT | IG |
| Col-125 | chr4 | 8048977 | +A | Intron |
| Col-125 | chr1 | 13074972 | +T | IG |
| Col-125 | chr1 | 2841386 | +A | IG |
| Col-125 | chr5 | 1755530 | +A | TE |
| Col-125 | chr5 | 5440248 | +T | IG |
| Col-125 | chr3 | 7274992 | +A | IG |
| Col-125 | chr4 | 5447587 | +AT | IG |
| Col-125 | chr3 | 4890747 | +T | IG |
| Col-125 | chr1 | 21731607 | +A | Intron |
| Col-125 | chr5 | 13189401 | +T | TE |
| Col-125 | chr3 | 8717736 | +A | Intron |
| Col-125 | chr4 | 17589123 | +T | IG |
| Col-125 | chr2 | 8484203 | +T | UTR |
| Col-125 | chr1 | 11046309 | +TA | IG |
| Col-125 | chr5 | 22707139 | +A | IG |
| Col-125 | chr1 | 18397686 | +AT | TE |
| Col-125 | chr5 | 11222898 | +A | TE |
| Col-125 | chr1 | 15607970 | +A | IG |
| nrpe-125 | chr5 | 18081396 | +A | TE |
| nrpe-125 | chr4 | 1769993 | +A | TE |
| nrpe-125 | chr2 | 8361910 | +AG | UTR |
| nrpe-125 | chr5 | 5262559 | +AG | Coding |
| nrpe-125 | chr1 | 6734196 | +A | Intron |
| nrpe-125 | chr2 | 10178219 | +T | Intron |
| nrpe-125 | chr1 | 3428045 | +ATTC | pseudogene |
| nrpe-125 | chr1 | 28314244 | +T | Intron |
| nrpe-125 | chr5 | 16914757 | +A | IG |
| nrpe-125 | chr2 | 3320263 | +G | IG |
| nrpe-125 | chr3 | 10619281 | +AT | IG |
| nrpe-125 | chr1 | 10624909 | +T | TE |
| nrpe-125 | chr5 | 12679237 | +C | IG |
| nrpe-125 | chr3 | 22962430 | +T | TE |
| nrpe-125 | chr1 | 7272158 | +A | IG |
| nrpe-125 | chr3 | 12630985 | +A | IG |
| nrpe-125 | chr4 | 1357881 | +T | TE |
| nrpe-125 | chr5 | 22276561 | +A | UTR |
| nrpe-125 | chr5 | 25134742 | +AG | UTR |
| nrpe-125 | chr4 | 9188547 | +C | IG |
| nrpe-125 | chr4 | 1443640 | +A | TE |
| nrpe-125 | chr4 | 3872562 | +AG | TE |
| nrpe-125 | chr2 | 15168955 | +T | Intron |
| ddc-125 | chr1 | 6334809 | +T | IG |
| ddc-125 | chr5 | 20005920 | +T | IG |
| ddc-125 | chr4 | 4355435 | +GT | IG |
| ddc-125 | chr5 | 10999266 | +A | TE |
| ddc-125 | chr5 | 12305003 | +AT | TE |
| ddc-125 | chr2 | 12520676 | +TA | TE |
| ddc-125 | chr1 | 1293802 | +AT | UTR |
| ddc-125 | chr3 | 14918949 | +A | TE |
| ddc-125 | chr5 | 1034946 | +T | UTR |
| ddc-125 | chr5 | 11367714 | +T | TE |
| ddc-125 | chr1 | 22165321 | +G | TE |
| ddc-125 | chr3 | 6761473 | +T | IG |
| ddc-125 | chr5 | 8731904 | +T | IG |
| ddc-125 | chr1 | 5073465 | +A | Intron |
| ddc-125 | chr5 | 16315 | +A | IG |
| ddc-125 | chr1 | 5090615 | +ACC | IG |
| ddc-125 | chr3 | 10692100 | +A | TE |
| ddc-125 | chr5 | 16369959 | +A | IG |
| ddc-125 | chr2 | 8115844 | +A | Intron |
| ddc-125 | chr1 | 23947190 | +TCATTTCTTAA | TE |
| ddc-150 | chr1 | 5090615 | +ACC | IG |
| ddc-150 | chr2 | 7096640 | +A | IG |
| ddc-150 | chr5 | 23637362 | +T | IG |
| ddc-150 | chr3 | 13825398 | +T | TE |
| ddc-150 | chr5 | 11367714 | +T | TE |
| ddc-150 | chr3 | 16999832 | +C | Intron |
| ddc-150 | chr4 | 10085286 | +T | Intron |
| ddc-150 | chr2 | 8115844 | +A | Intron |
| ddc-150 | chr2 | 2770073 | +T | TE |
| ddc-150 | chr1 | 12885498 | +T | TE |
| ddc-150 | chr1 | 10326726 | +TC | UTR |
| ddc-150 | chr3 | 14103573 | +A | TE |
| ddc-150 | chr4 | 4355435 | +GT | IG |
| ddc-150 | chr5 | 23598558 | +A | UTR |
| ddc-150 | chr1 | 1293802 | +AT | UTR |
| ddc-150 | chr4 | 985005 | +T | TE |
| ddc-150 | chr5 | 20005920 | +T | IG |
| ddc-175 | chr5 | 8731904 | +T | IG |
| ddc-175 | chr2 | 2770073 | +T | TE |
| ddc-175 | chr2 | 3845329 | +T | TE |
| ddc-175 | chr1 | 23947190 | +TCATTTCTTAA | TE |
| ddc-175 | chr2 | 12877696 | +A | TE |
| ddc-175 | chr4 | 1731139 | +T | IG |
| ddc-175 | chr3 | 6761473 | +T | IG |
| ddc-175 | chr5 | 1073771 | +T | IG |
| ddc-175 | chr5 | 967731 | +A | IG |
| ddc-175 | chr5 | 16369959 | +A | IG |
| ddc-175 | chr5 | 6570227 | +T | Intron |
| ddc-175 | chr4 | 4428184 | +G | TE |
| ddc-175 | chr4 | 4355435 | +GT | IG |
| ddc-175 | chr2 | 7096640 | +A | IG |
| ddc-175 | chr1 | 1293802 | +AT | UTR |
| ddc-175 | chr5 | 11367714 | +T | TE |
| ddc-175 | chr4 | 15589780 | +A | UTR |
| ddc-175 | chr1 | 10326726 | +TC | UTR |
| ddc-175 | chr3 | 14103573 | +A | TE |
| ddc-175 | chr3 | 14225229 | +GA | TE |
| ddc-175 | chr2 | 2561617 | +C | IG |
| ddc-175 | chr2 | 2763562 | +CAT | UTR |
| ddc-175 | chr2 | 19019943 | +ATGTG | IG |
| ddc-175 | chr1 | 12885498 | +T | TE |
|  |  |  |  |  |

Table S4. Number of mutations and their distributions among functional classes within the genome of 2^nd^ batch of SAD and SUT cells

| SNP | Col |  | nrpe |  |  | ddc |
| --- | --- | --- | --- | --- | --- | --- |
| NaCl (mM) | 0 | 150 | 0 | 150 | 0 | 150 |
| Coding | 14 | 7 | 2 | 4 | 4 | 14 |
| IG | 16 | 22 | 3 | 7 | 7 | 14 |
| Intron | 12 | 7 | 3 | 0 | 0 | 6 |
| TE | 28 | 34 | 8 | 8 | 9 | 18 |
| UTR | 3 | 0 | 0 | 0 | 1 | 2 |
| ncRNA | 2 | 0 | 0 | 1 | 0 | 0 |
| pseudogene | 1 | 1 | 0 | 0 | 0 | 1 |
| Total Number | 76 | 71 | 16 | 20 | 21 | 55 |
| MR (X10^-10^) | 35.4 | 32.2 | 7.49 | 9.24 | 9.77 | 30.2 |
| INDEL |  |  |  |  |  |  |
| Coding | 1 | 1 | 0 | 0 | 0 | 0 |
| IG | 6 | 2 | 1 | 3 | 0 | 1 |
| Intron | 0 | 0 | 0 | 1 | 1 | 1 |
| TE | 1 | 2 | 0 | 0 | 0 | 2 |
| UTR | 0 | 0 | 1 | 0 | 0 | 0 |
| ncRNA | 0 | 0 | 0 | 0 | 0 | 0 |
| pseudogene | 0 | 0 | 0 | 0 | 0 | 0 |
| Total | 8 | 5 | 2 | 4 | 1 | 4 |
| MR (X10-10) | 3.72 | 2.27 | 0.94 | 1.85 | 0.47 | 2.19 |

Table S5 A list of total coding region mutations in the first batch of SUT and SAD cells.

| GeneID | Mutation variant | | Gene function description | | |
| --- | --- | --- | --- | --- | --- |
| Col-0 |  | |  | | |
| AT1G31640 | synonymous | | AGAMOUS-like 92 | | |
| AT1G50980 | missense | | F-box/RNI-like/FBD-like domains-containing protein | | |
| **AT1G57630** | missense | | Toll-Interleukin-Resistance (TIR) domain family protein | | |
| **AT1G71330** | missense | | non-intrinsic ABC protein 5 | | |
| **AT1G73080** | missense | | PEP1 receptor 1 | | |
| AT1G79410 | synonymous | | organic cation/carnitine transporter5 | | |
| **AT2G14820** | missense | | Phototropic-responsive NPH3 family protein | | |
| AT3G47180 | missense | | RING/U-box superfamily protein | | |
| **AT3G47960** | splice_region | | Major facilitator superfamily protein | | |
| **AT3G48730** | missense | | glutamate-1-semialdehyde 2,1-aminomutase 2 | | |
| AT4G01330 | missense | | Protein kinase superfamily protein | | |
| AT4G11670 | synonymous | | Protein of unknown function (DUF810) | | |
| AT4G16144 | splice_region | | associated molecule with the SH3 domain of STAM 3 | | |
| **AT4G16600** | missense | | Nucleotide-diphospho-sugar transferases superfamily protein | | |
| AT4G23950 | synonymous | | Galactose-binding protein | | |
|  |  | |  | | |
| Col-125 |  | |  | | |
| **AT1G12780** | missense | | UDP-D-glucose/UDP-D-galactose 4-epimerase 1 | | |
| AT1G15680 | synonymous | | F-box family protein | | |
| **AT1G26770** | missense | | expansin A10 | | |
| **AT1G35617** | missense | |  | | |
| AT1G40104 | missense | |  | | |
| AT1G47578 | missense | | Biotin/lipoate A/B protein ligase family | | |
| AT1G70610 | missense | | transporter associated with antigen processing protein 1 | | |
| **AT2G27090** | missens | | Protein of unknown function (DUF630 and DUF632) | | |
| AT2G37280 | splice_region | | pleiotropic drug resistance 5 | | |
| **AT4G23600** | synonymous | | Tyrosine transaminase family protein | | |
| AT4G26610 | missense | | D6 protein kinase like 1 | | |
| AT5G17410 | missense | | Spc97 / Spc98 family of spindle pole body (SBP) component | | |
| AT5G17410 | splice_region | | Spc97 / Spc98 family of spindle pole body (SBP) component | | |
|  |  | |  | | |
| nrpe-0 |  | |  | | |
| **AT1G04770** | synonymous | | Tetratricopeptide repeat (TPR)-like superfamily protein | | |
| AT1G13730 | missense | | Nuclear transport factor 2 (NTF2) family protein with RNA binding (RRM-RBD-RNP motifs) domain | | |
| **AT1G30200** | synonymous | | F-box family protein | | |
| **AT1G30925** | synonymous | | phospholipase Cs | | |
| **AT1G52400** | synonymous | | beta glucosidase 18 | | |
| AT1G64620 | missense | | Dof-type zinc finger DNA-binding family protein | | |
| AT1G76370 | missense | | Protein kinase superfamily protein | | |
| **AT1G77440** | missense | | 20S proteasome beta subunit C2 | | |
| AT2G04540 | missense | | Beta-ketoacyl synthase | | |
| AT2G13680 | missense | | callose synthase 5 | | |
| AT2G31650 | missense | | homologue of trithorax | | |
| AT2G36380 | missense | | pleiotropic drug resistance 6 | | |
| AT2G47410 | missense | | WD40/YVTN repeat-like-containing domain;Bromodomain | | |
| **AT2G48080** | synonymous | | oxidoreductase, 2OG-Fe(II) oxygenase family protein | | |
| **AT3G12145** | synonymous | | Leucine-rich repeat (LRR) family protein | | |
| AT3G22345 | stop_gained | |  | | |
| AT3G27473 | missense | | Cysteine/Histidine-rich C1 domain family protein | | |
| AT3G48410 | splice_region | | alpha/beta-Hydrolases superfamily protein | | |
| **AT3G63450** | missense | | RNA-binding (RRM/RBD/RNP motifs) family protein | | |
| AT3G63500 | missense | | Protein of unknown function (DUF1423) | | |
| AT4G10070 | missense | | KH domain-containing protein | | |
| **AT4G27720** | missens | | Major facilitator superfamily protein | | |
| AT5G02930 | missense | | F-box/RNI-like superfamily protein | | |
| **AT5G25190** | missense | | Integrase-type DNA-binding superfamily protein | | |
| AT5G39290 | missense | | expansin A26 | | |
| **AT5G54190** | missense | | protochlorophyllide oxidoreductase A | | |
|  |  | |  | | |
| nrpe-125 |  | |  | | |
| **AT1G06120** | splice_region | | Fatty acid desaturase family protein | | |
| AT1G13910 | missense | | Leucine-rich repeat (LRR) family protein | | |
| AT1G13910 | splice_region | | Leucine-rich repeat (LRR) family protein | | |
| **AT1G28440** | missense | | HAESA-like 1 | | |
| AT1G30570 | missense | | hercules receptor kinase 2 | | |
| **AT1G33811** | missense | | GDSL-like Lipase/Acylhydrolase superfamily protein | | |
| AT1G43260 | synonymous | | hAT transposon superfamily protein | | |
| **AT1G50240** | stop_gained | | Protein kinase family protein with ARM repeat domain | | |
| AT1G60140 | stop_lost | | trehalose phosphate synthase | | |
| AT1G62600 | synonymous | | Flavin-binding monooxygenase family protein | | |
| AT1G67550 | synonymous | | urease | | |
| AT1G68710 | missense | | ATPase E1-E2 type family protein / haloacid dehalogenase-like hydrolase family protein | | |
| AT1G79920 | missense | | Heat shock protein 70 (Hsp 70) family protein | | |
| AT2G07280 | missense | |  | | |
| AT2G17580 | synonymous | | Polynucleotide adenylyltransferase family protein | | |
| **AT2G22250** | synonymous | | aspartate aminotransferase | | |
| **AT3G02370** | missense |  | | |  |
| **AT3G10300** | missense | | Calcium-binding EF-hand family protein | | |
| **AT3G15115** | missense | |  | | |
| AT3G19220 | missense | | protein disulfide isomerases | | |
| **AT3G20530** | synonymous | | Protein kinase superfamily protein | | |
| AT3G44620 | missense | | protein tyrosine phosphatases;protein tyrosine phosphatases | | |
| **AT3G47350** | splice_region | | hydroxysteroid dehydrogenase 2 | | |
| **AT3G51910** | missense | | heat shock transcription factor A7A | | |
| AT3G58050 | synonymous | |  | | |
| **AT3G61470** | missense | | photosystem I light harvesting complex gene 2 | | |
| AT4G02900 | missense | | ERD (early-responsive to dehydration stress) family protein | | |
| **AT4G03030** | missense | | Galactose oxidase/kelch repeat superfamily protein | | |
| AT4G03090 | missense | | sequence-specific DNA binding;sequence-specific DNA binding transcription factors | | |
| **AT4G03200** | 5_prime_UTR_premature_start_codon_gain | | catalytics | | |
| **AT4G03200** | splice_region | | catalytics | | |
| **AT4G03200** | stop_gained | | catalytics | | |
| **AT4G03415** | 5_prime_UTR_premature_start_codon_gain | | Protein phosphatase 2C family protein | | |
| **AT4G10350** | missense | | NAC domain containing protein 70 | | |
| AT4G20700 | synonymous | | Protein of unknown function (DUF1204) | | |
| **AT4G35790** | missense | | phospholipase D delta | | |
| **AT4G39950** | missense | | cytochrome P450, family 79, subfamily B, polypeptide 2 | | |
| AT5G09870 | missense | | cellulose synthase 5 | | |
| AT5G10550 | synonymous | | global transcription factor group E2 | | |
| AT5G16110 | disruptive_inframe_insertion | | | | |
| **AT5G22000** | splice_donor | | | RING-H2 group F2A | |
| AT5G27330 | missense | | | Prefoldin chaperone subunit family protein | |
| AT5G36740 | missense | | | Acyl-CoA N-acyltransferase with RING/FYVE/PHD-type zinc finger protein | |
| AT5G42750 | missense | | | BRI1 kinase inhibitor 1 | |
| AT5G54330 | missense | | | Protein of unknown function (DUF295) | |
| AT5G57710 | missense | | | Double Clp-N motif-containing P-loop nucleoside triphosphate hydrolases superfamily protein | |
| **AT5G59670** | missense | | | Leucine-rich repeat protein kinase family protein | |
|  |  | | |  | |
| ddc-0 |  | | |  | |
| **AT1G03010** | missense | | | Phototropic-responsive NPH3 family protein | |
| AT1G07910 | missense | | | RNAligase | |
| **AT1G26270** | synonymous | | | Phosphatidylinositol 3- and 4-kinase family protein | |
| AT1G40104 | splice_region | | |  | |
| **AT1G53070** | synonymous | | | Legume lectin family protein | |
| **AT1G70370** | missense | | | polygalacturonase 2 | |
| AT2G01820 | missense | | | Leucine-rich repeat protein kinase family protein | |
| **AT2G03840** | missense | | | tetraspanin13 | |
| **AT2G26800** | missense | | | Aldolase superfamily protein | |
| **AT2G26800** | splice_region | | | Aldolase superfamily protein | |
| **AT2G36770** | missense | | | UDP-Glycosyltransferase superfamily protein | |
| AT3G46120 | missense | | | purple acid phosphatase 19 | |
| **AT3G55210** | missense | | | NAC domain containing protein 63 | |
| AT4G05410 | missense | | | Transducin/WD40 repeat-like superfamily protein | |
| **AT4G21680** | missense | | | NITRATE TRANSPORTER 1.8 | |
| **AT4G26970** | missense | | | aconitase 2 | |
| AT4G27430 | missense | | | COP1-interacting protein 7 | |
| **AT5G02630** | missense | | | Lung seven transmembrane receptor family protein | |
| AT5G27030 | missense | | | TOPLESS-related 3 | |
| **AT5G57590** | synonymous | | | adenosylmethionine-8-amino-7-oxononanoate transaminases | |
| **AT5G64490** | missense | | | ARM repeat superfamily protein | |
|  |  | | |  | |
| ddc-125 |  | | |  | |
| AT1G04190 | missense | | | Tetratricopeptide repeat (TPR)-like superfamily protein | |
| AT1G07910 | missense | | | RNAligase | |
| AT1G12040 | synonymous | | | leucine-rich repeat/extensin 1 | |
| **AT1G26270** | synonymous | | | Phosphatidylinositol 3- and 4-kinase family protein | |
| AT1G29740 | missense | | | Leucine-rich repeat transmembrane protein kinase | |
| **AT1G49010** | stop_gained | | | Duplicated homeodomain-like superfamily protein | |
| **AT1G53070** | synonymous_ | | | Legume lectin family protein | |
| AT1G59890 | missense | | | SIN3-like 5 | |
| **AT1G70370** | missense | | | polygalacturonase 2 | |
| **AT1G74070** | stop_gained | | | Cyclophilin-like peptidyl-prolyl cis-trans isomerase family protein | |
| **AT1G76160** | missense_ | | | SKU5 similar 5 | |
| **AT1G77440** | missense | | | 20S proteasome beta subunit C2 | |
| AT1G80040 | missense | | |  | |
| AT2G03250 | synonymous | | | EXS (ERD1/XPR1/SYG1) family protein | |
| **AT2G03840** | missense | | | tetraspanin13 | |
| **AT2G22430** | stop_gained | | | homeobox protein 6 | |
| AT2G26910 | synonymous | | | pleiotropic drug resistance 4 | |
| **AT2G34300** | missense | | | S-adenosyl-L-methionine-dependent methyltransferases superfamily protein | |
| **AT2G42790** | missense | | | citrate synthase 3 | |
| AT2G43290 | missense_ | | | Calcium-binding EF-hand family protein | |
| **AT3G02230** | missense | | | reversibly glycosylated polypeptide 1 | |
| **AT3G10240** | stop_gained | | | F-box and associated interaction domains-containing protein | |
| AT3G13220 | missense | | | ABC-2 type transporter family protein | |
| **AT3G20160** | synonymous | | | Terpenoid synthases superfamily protein | |
| **AT3G21760** | synonymous | | | UDP-Glycosyltransferase superfamily protein | |
| **AT3G28958** | missense | | | Cupredoxin superfamily protein | |
| AT3G44230 | missense | | |  | |
| **AT3G44770** | missense | | | Protein of unknown function (DUF626) | |
| AT3G46120 | missense | | | purple acid phosphatase 19 | |
| **AT3G55210** | missense | | | NAC domain containing protein 63 | |
| **AT3G55410** | stop_gained | | | 2-oxoglutarate dehydrogenase, E1 component | |
| **AT3G56400** | synonymous | | | WRKY DNA-binding protein 70 | |
| **AT3G57600** | synonymous | | | Integrase-type DNA-binding superfamily protein | |
| **AT3G59850** | synonymous | | | Pectin lyase-like superfamily protein | |
| **AT3G60670** | missense | | | PLATZ transcription factor family protein | |
| AT3G61700 | missense | | | Plant protein 1589 of unknown function | |
| AT4G05410 | missense | | | Transducin/WD40 repeat-like superfamily protein | |
| AT4G13380 | stop_gained | | | Heavy metal transport/detoxification superfamily protein | |
| **AT4G21680** | missense_variant | | | NITRATE TRANSPORTER 1.8 | |
| **AT4G26530** | stop_gained | | | Aldolase superfamily protein | |
| **AT4G26970** | missense | | | aconitase 2 | |
| AT4G27430 | missense | | | COP1-interacting protein 7 | |
| **AT4G36210** | splice_acceptor | | | Protein of unknown function (DUF726) | |
| **AT5G01200** | missense | | | Duplicated homeodomain-like superfamily protein | |
| **AT5G02630** | missense | | | Lung seven transmembrane receptor family protein | |
| **AT5G03860** | splice_region | | | malate synthase | |
| **AT5G09930** | missense | | | ABC transporter family protein | |
| AT5G16420 | missense | | | Pentatricopeptide repeat (PPR-like) superfamily protein | |
| **AT5G26220** | missense | | | ChaC-like family protein | |
| AT5G27030 | missense | | | TOPLESS-related 3 | |
| **AT5G37810** | missense | | | NOD26-like intrinsic protein 4;1 | |
| AT5G47940 | synonymous | | |  | |
| **AT5G59090** | missense | | | subtilase 4.12 | |
| AT5G61760 | missense | | | inositol polyphosphate kinase 2 beta | |
| **AT5G64490** | missense | | | ARM repeat superfamily protein | |
| AT5G65170 | missense | | | VQ motif-containing protein | |
| **AT5G66030** | missense | | | Golgi-localized GRIP domain-containing protein | |
|  |  | | |  | |
| ddc-150 |  | | |  | |
| AT1G04190 | missense | | | Tetratricopeptide repeat (TPR)-like superfamily protein | |
| AT1G07615 | missense | | | GTP-binding protein Obg/CgtA | |
| AT1G07910 | missense | | | RNAligase | |
| **AT1G19460** | missense | | | Galactose oxidase/kelch repeat superfamily protein | |
| AT1G29740 | missense | | | Leucine-rich repeat transmembrane protein kinase | |
| **AT1G53070** | synonymous | | | Legume lectin family protein | |
| AT1G59890 | missense | | | SIN3-like 5 | |
| **AT1G70370** | missense | | | polygalacturonase 2 | |
| **AT1G76160** | missense | | | SKU5 similar 5 | |
| **AT1G77440** | missense | | | 20S proteasome beta subunit C2 | |
| AT1G80040 | missense | | |  | |
| AT2G01008 | synonymous | | |  | |
| **AT2G03840** | missense | | | tetraspanin13 | |
| **AT2G24280** | missense | | | alpha/beta-Hydrolases superfamily protein | |
| AT2G26910 | synonymous | | | pleiotropic drug resistance 4 | |
| **AT2G42790** | missense | | | citrate synthase 3 | |
| **AT3G02230** | missense | | | reversibly glycosylated polypeptide 1 | |
| **AT3G10240** | stop_gained | | | F-box and associated interaction domains-containing protein | |
| AT3G13220 | missense | | | ABC-2 type transporter family protein | |
| AT3G19330 | missense | | | Protein of unknown function (DUF677) | |
| **AT3G20160** | synonymous | | | Terpenoid synthases superfamily protein | |
| **AT3G21760** | synonymous | | | UDP-Glycosyltransferase superfamily protein | |
| **AT3G28958** | missense | | | Cupredoxin superfamily protein | |
| AT3G41762 | missense_ | | |  | |
| AT3G44230 | missense | | |  | |
| **AT3G44770** | missense | | | Protein of unknown function (DUF626) | |
| AT3G46120 | missense | | | purple acid phosphatase 19 | |
| **AT3G55210** | missense | | | NAC domain containing protein 63 | |
| **AT3G55410** | stop_gained | | | 2-oxoglutarate dehydrogenase, E1 component | |
| **AT3G56400** | synonymous | | | WRKY DNA-binding protein 70 | |
| **AT3G59850** | synonymous | | | Pectin lyase-like superfamily protein | |
| **AT3G60670** | missense | | | PLATZ transcription factor family protein | |
| AT3G61700 | missense | | | Plant protein 1589 of unknown function | |
| AT4G05410 | missense | | | Transducin/WD40 repeat-like superfamily protein | |
| **AT4G21680** | missense_ | | | NITRATE TRANSPORTER 1.8 | |
| **AT4G26970** | missense | | | aconitase 2 | |
| AT4G27060 | missense | | | ARM repeat superfamily protein | |
| **AT4G35180** | stop_gained | | | LYS/HIS transporter 7 | |
| **AT4G36210** | splice_acceptor_ | | | Protein of unknown function (DUF726) | |
| **AT5G09930** | missense_ | | | ABC transporter family protein | |
| AT5G16420 | missense | | | Pentatricopeptide repeat (PPR-like) superfamily protein | |
| **AT5G26180** | missense | | | S-adenosyl-L-methionine-dependent methyltransferases superfamily protein | |
| **AT5G26220** | missense | | | ChaC-like family protein | |
| AT5G27030 | missense | | | TOPLESS-related 3 | |
| AT5G37380 | 5_prime_UTR_premature_start_codon_gain | | | Chaperone DnaJ-domain superfamily protein | |
| **AT5G37810** | missense | | | NOD26-like intrinsic protein 4;1 | |
| AT5G42920 | missense | | | THO complex, subunit 5 | |
| AT5G47940 | synonymous | | |  | |
| AT5G61760 | missense | | | inositol polyphosphate kinase 2 beta | |
| **AT5G64490** | missense | | | ARM repeat superfamily protein | |
| AT5G65170 | missense | | | VQ motif-containing protein | |
| **AT5G66030** | missense | | | Golgi-localized GRIP domain-containing protein | |
|  |  | | |  | |
| ddc-175 |  | | |  | |
| AT1G04190 | missense | | | Tetratricopeptide repeat (TPR)-like superfamily protein | |
| AT1G07615 | missense | | | GTP-binding protein Obg/CgtA | |
| AT1G07910 | missense | | | RNAligase | |
| AT1G12040 | synonymous | | | leucine-rich repeat/extensin 1 | |
| **AT1G19460** | missense | | | Galactose oxidase/kelch repeat superfamily protein | |
| **AT1G26270** | synonymous | | | Phosphatidylinositol 3- and 4-kinase family protein | |
| AT1G29740 | missense | | | Leucine-rich repeat transmembrane protein kinase | |
| **AT1G53070** | synonymous | | | Legume lectin family protein | |
| AT1G59890 | missense | | | SIN3-like 5 | |
| **AT1G70370** | missense | | | polygalacturonase 2 | |
| **AT1G74070** | stop_gained | | | Cyclophilin-like peptidyl-prolyl cis-trans isomerase family protein | |
| **AT1G76160** | missense | | | SKU5 similar 5 | |
| **AT1G77440** | missense | | | 20S proteasome beta subunit C2 | |
| AT1G80040 | missense | | |  | |
| AT2G03250 | synonymous | | | EXS (ERD1/XPR1/SYG1) family protein | |
| **AT2G03840** | missense | | | tetraspanin13 | |
| AT2G26910 | synonymous | | | pleiotropic drug resistance 4 | |
| **AT2G42790** | missense | | | citrate synthase 3 | |
| AT2G43290 | missense | | | Calcium-binding EF-hand family protein | |
| **AT3G02230** | missense | | | reversibly glycosylated polypeptide 1 | |
| AT3G06450 | stop_gained | | | HCO3- transporter family | |
| **AT3G10240** | stop_gained | | | F-box and associated interaction domains-containing protein | |
| AT3G13220 | missense | | | ABC-2 type transporter family protein | |
| AT3G19330 | missense | | | Protein of unknown function (DUF677) | |
| **AT3G20160** | synonymous | | | Terpenoid synthases superfamily protein | |
| **AT3G21760** | synonymous | | | UDP-Glycosyltransferase superfamily protein | |
| **AT3G28958** | missense | | | Cupredoxin superfamily protein | |
| AT3G41762 | missense | | |  | |
| AT3G44230 | missense | | |  | |
| **AT3G44770** | missense | | | Protein of unknown function (DUF626) | |
| AT3G46120 | missense | | | purple acid phosphatase 19 | |
| **AT3G51560** | synonymous | | | Disease resistance protein (TIR-NBS-LRR class) family | |
| **AT3G55210** | missense | | | NAC domain containing protein 63 | |
| **AT3G55410** | stop_gained | | | 2-oxoglutarate dehydrogenase, E1 component | |
| **AT3G56400** | synonymous | | | WRKY DNA-binding protein 70 | |
| **AT3G57600** | synonymous | | | Integrase-type DNA-binding superfamily protein | |
| **AT3G60670** | missense | | | PLATZ transcription factor family protein | |
| AT3G61700 | missense | | | Plant protein 1589 of unknown function | |
| AT4G05410 | missense | | | Transducin/WD40 repeat-like superfamily protein | |
| **AT4G21680** | missense | | | NITRATE TRANSPORTER 1.8 | |
| **AT4G26530** | stop_gained | | | Aldolase superfamily protein | |
| **AT4G26970** | missense | | | aconitase 2 | |
| AT4G27060 | missense | | | ARM repeat superfamily protein | |
| AT4G27430 | missense | | | COP1-interacting protein 7 | |
| **AT4G35180** | stop_gained | | | LYS/HIS transporter 7 | |
| **AT4G36210** | splice_acceptor | | | Protein of unknown function (DUF726) | |
| **AT5G02630** | missense | | | Lung seven transmembrane receptor family protein | |
| AT5G16420 | missense | | | Pentatricopeptide repeat (PPR-like) superfamily protein | |
| **AT5G26180** | missense | | | S-adenosyl-L-methionine-dependent methyltransferases superfamily protein | |
| **AT5G26220** | missense | | | ChaC-like family protein | |
| AT5G27030 | missense | | | TOPLESS-related 3 | |
| **AT5G37810** | missense | | | NOD26-like intrinsic protein 4;1 | |
| AT5G47940 | synonymous | | |  | |
| **AT5G52170** | missense | | | homeodomain GLABROUS 7 | |
| **AT5G59090** | missense | | | subtilase 4.12 | |
| AT5G61760 | missense | | | inositol polyphosphate kinase 2 beta | |
| **AT5G64490** | missense | | | ARM repeat superfamily protein | |
| AT5G65170 | missense | | | VQ motif-containing protein | |

Note: Genes highlighted in bold are stress responsive genes
